# Association Between Colonoscopy Withdrawal Time and Adenoma Detection: Evidence of a Continuous Dose–Response Relationship

**DOI:** 10.3390/diagnostics16172693

**Published:** 2026-08-24

**Authors:** Majd Khader, Rimon Artoul, Fadi Abu Baker, Jorge-Shmuel Delgado, Tali Braun, Yudit Meltzer, Ronit Ahdut HaCohen, Rawi Hazzan

**Affiliations:** 1 Gastroenterology Institute, Assuta Medical Center, Beer Sheva 8489507, Israel; 2 Faculty of Health Sciences, Ben-Gurion University of the Negev, Beer Sheva 8410501, Israel; 3 Geriatrics Department, Barzilai Medical Center, Ashkelon 7830604, Israel; 4 Department of Gastroenterology and Hepatology, Hillel Yaffe Medical Center, Hadera 38100, Israel; 5 Faculty of Medicine, Technion, Haifa 3200003, Israel; 6 Maccabi Health Services South District, Beer Sheva 8448102, Israel; 7 Department of Science, David Yellin Academic College of Education, Jerusalem 9103501, Israel; 8 Liver Clinic, Clalit Health Service, Nof Haglil 1750400, Israel; 9 Azrieli Faculty of Medicine, Bar-Ilan University, Safad 1311502, Israel

**Keywords:** colonoscopy, adenoma detection rate (ADR), withdrawal time, quality indicators, colorectal cancer prevention

## Abstract

**Background/Objectives:** Colonoscopy withdrawal time is an established quality indicator, but ongoing debate exists regarding whether its relationship with adenoma detection follows a fixed threshold or a continuous dose–response pattern. **Methods:** We retrospectively analyzed 31,780 colonoscopies from a multicenter endoscopy database to evaluate the relationship between withdrawal duration and lesion detection. Withdrawal time was assessed as both a continuous and categorical variable, and adenoma detection rate (ADR) and polyp detection rate (PDR) were examined across quartiles and clinically relevant intervals. **Results:** ADR increased progressively from 7.72% in the shortest withdrawal-time quartile to 36.47% in the longest quartile, while PDR increased from 21.22% to 67.86% (both *p* for trend <0.001). In the multivariable analysis adjusted for age, sex, and bowel-preparation quality, each additional minute of withdrawal time was independently associated with higher odds of adenoma detection (adjusted OR 1.14, 95% CI 1.13–1.15; *p* < 0.001). Although the confidence interval was narrow, reflecting the large sample size, the effect was clinically appreciable: model-predicted adenoma detection increased from 13.6% at six minutes to 21.0% at eight minutes and 28.2% at ten minutes, corresponding to approximately seven and fifteen additional adenoma-positive examinations per hundred procedures, respectively. The association remained consistent across age and sex strata. Receiver operating characteristic analysis showed moderate discrimination (area under the curve 0.701), with a Youden-optimal region of approximately 7 to 8 min. Because recorded withdrawal time incorporates interventional time, the principal analysis of inspection effort was conducted at the level of the endoscopist, using withdrawal time measured exclusively in the 14,611 examinations in which no polyp was detected and no tissue was sampled. Among 107 endoscopists contributing 26,793 procedures, inspection time was associated with adenoma detection (Spearman ρ = 0.46; *p* < 0.001), corresponding to an absolute increase of approximately 1.5 percentage points per additional minute, with attenuation of the gradient beyond approximately seven minutes. **Conclusions:** The procedure-level findings above describe the association with recorded withdrawal duration as captured in routine practice and are reported as supportive rather than as estimates of inspection effort. Together these analyses indicate that inspection time is associated with adenoma detection in a graded manner extending beyond the historical 6 min benchmark.

## 1. Introduction

Colonoscopy is the cornerstone of colorectal cancer (CRC) prevention, enabling the detection and removal of premalignant lesions before progression to invasive malignancy [1,2,3,4,5]. The effectiveness of colonoscopy, however, is highly dependent on procedural quality [6,7,8]. Among established quality indicators, adenoma detection rate (ADR) has emerged as the most clinically relevant benchmark, demonstrating a strong inverse association with the risk of interval colorectal cancer and CRC-related mortality [9,10,11].

Withdrawal time, which reflects the duration of mucosal inspection during scope withdrawal, is one of the few modifiable procedural factors consistently associated with ADR [12,13,14]. Several studies reported improved adenoma detection among endoscopists with mean withdrawal times of at least six minutes, leading professional societies to incorporate a minimum 6 min withdrawal duration into quality guidelines. This threshold was revised upward in 2024, when the ASGE/ACG quality indicators recommended a mean withdrawal time of at least 8 min in normal colonoscopies; the 6 min figure is therefore referred to throughout this report as the historical benchmark [13,15,16,17]. Nevertheless, the biological and clinical significance of withdrawal time beyond this threshold remains incompletely understood.

Although longer withdrawal durations have generally been associated with higher lesion detection rates [15,16,17,18,19], several important questions remain unresolved. First, it is unclear whether the relationship between withdrawal duration and adenoma detection follows a threshold effect or a continuous dose–response pattern. Second, the withdrawal duration associated with optimal detection performance has not been definitively established. Third, whether a plateau in detection efficiency is reached within clinically achievable withdrawal times remains uncertain. Finally, data evaluating the consistency of these associations across demographic risk strata are limited.

Addressing these questions is clinically important because withdrawal time represents a readily modifiable component of colonoscopy quality [13,15,16,17]. If lesion detection continues to improve beyond currently accepted procedural benchmarks, existing quality standards may underestimate the withdrawal duration required to maximize neoplasia detection.

Therefore, we performed a large-scale analysis of 31,780 colonoscopies to comprehensively evaluate the relationship between withdrawal duration and lesion detection. We specifically investigated whether withdrawal time shows a dose-dependent association with adenoma detection and assessed whether an optimal withdrawal threshold exists. We examined whether a detection plateau emerges as withdrawal intervals are progressively prolonged. We evaluated the stability of these associations across age and sex subgroups. In addition, because recorded withdrawal times incorporate time spent on therapeutic maneuvers, we evaluated the association at the level of the individual endoscopist using inspection time measured exclusively in examinations in which no polyp was detected and no tissue was sampled, thereby separating the measurement of inspection effort from the detection of lesions.

## 2. Methods

### 2.1. Study Design and Population

This retrospective observational study analyzed consecutive colonoscopies performed during 2025 across seven gastroenterology units of Assuta Medical Centers (Ra’anana, Haifa, Ramat HaChayal, Holon, Ashdod, Jerusalem, and Beer Sheva). Procedures were eligible for inclusion if they had (i) an automatically recorded cecal-intubation and rectal-withdrawal timestamp permitting calculation of withdrawal time, (ii) a linked endoscopy report with documented lesion-detection outcomes, (iii) a documented Boston Bowel-Preparation Scale (BBPS) score, and (iv) complete demographic data. Procedures were excluded if withdrawal time could not be derived from the video annotation, if the endoscopy report could not be linked to the procedure record, or if bowel-preparation quality or demographic data were missing. A total of 31,780 colonoscopies met these criteria and constituted the final analytic cohort (Supplementary Figure S1). The indication for each examination was recorded within the clinical endoscopy report as one or more structured presenting-complaint entries. These were classified hierarchically as positive fecal occult blood testing, polyp surveillance, family history of colorectal cancer, screening, or symptomatic and diagnostic presentation, in that order of precedence where more than one entry was present. An indication could be assigned in 25,593 of 31,780 procedures (80.5%); the remainder were recorded as other or unspecified and were retained as a separate category rather than excluded. Indication was included as a covariate in multivariable models, and a pre-specified analysis was additionally restricted to examinations performed for screening.

Bowel preparation was assessed using the Boston Bowel-Preparation Scale, with adequate preparation defined as a total score of at least 6 with no segment scoring below 2. Every procedure in the analytic cohort carried a total score between 6 and 9; no examination scored below 6, and none was excluded on the grounds of inadequate bowel preparation.

### 2.2. Colonoscopy Variables

Withdrawal time was defined as the interval between initiation of withdrawal from the cecum and completion of colonoscope withdrawal, as automatically recorded within the endoscopy reporting system. The recorded withdrawal time included time spent performing diagnostic and therapeutic interventions during withdrawal, including biopsy, polypectomy, and mucosal cleaning. For analytical purposes, withdrawal duration was evaluated both as a continuous variable and as categorized intervals. Two categorization strategies were applied. First, withdrawal duration was divided into quartiles to assess exposure–response relationships. Second, clinically relevant withdrawal-time categories were defined as <6 min, 6–8 min, 8–10 min, and >10 min. Additional non-linear analyses were performed using finer withdrawal intervals (0–4, 4–6, 6–8, 8–10, 10–15, and >15 min). The primary outcome was adenoma detection rate (ADR), defined as the proportion of colonoscopies in which at least one histologically confirmed adenoma was identified. The secondary outcome was polyp detection rate (PDR), defined as the proportion of examinations in which at least one colorectal polyp was detected.

The status of examinations without a confirmed cecal landmark was examined in detail. A cecal landmark was confirmed by the annotation system in 29,607 of 31,780 procedures (93.2%); in 1099 the cecal time was inferred by the system and in 1074 no cecal time was recorded. In these 2173 procedures the recorded withdrawal time was almost identical to the total procedure duration (median ratio 1.000, interquartile range 0.968 to 1.000, with 80.3% falling within sixty seconds of the total duration), whereas in procedures with a confirmed landmark the withdrawal time represented approximately half of the procedure (median ratio 0.520, interquartile range 0.405 to 0.627, with 0.3% within sixty seconds). Total procedure duration was, however, indistinguishable between the two groups (median 13.2 min in both). Failure to identify the cecal landmark therefore caused the withdrawal interval to default to approximately the whole procedure, systematically overestimating withdrawal time, rather than indicating a shorter or incomplete examination. Because this artifact affects the exposure, all principal and supportive analyses were repeated with the cohort restricted to procedures with a confirmed cecal landmark.

#### Endoscopist-Level Analysis and Sensitivity Analyses

Because the recorded withdrawal time incorporates time spent on biopsy, polypectomy, and mucosal cleaning, procedure-level associations between withdrawal duration and adenoma detection are susceptible to reverse causation. Adenoma detection cannot be evaluated in procedures from which interventional time has been removed, since histological confirmation requires tissue sampling. We therefore performed an endoscopist-level analysis modeled on the design of Barclay et al. [16] and on the formal definition of the withdrawal-time quality indicator adopted by the ASGE/ACG Task Force, in which average withdrawal time is measured in normal colonoscopies performed without biopsy sampling or polypectomy.

For each endoscopist, inspection time was defined as the mean withdrawal time across colonoscopies in which no polyp was detected and no tissue was sampled. Each endoscopist’s ADR and PDR were calculated across all of their procedures. Analyses were restricted to endoscopists contributing at least 100 procedures and at least 30 negative examinations. Associations between inspection time and detection rates were assessed using Pearson and Spearman correlation coefficients and by comparing detection rates across quartiles of inspection time. In a pre-specified sensitivity analysis, endoscopists with a mean inspection time below 3 min were excluded, as such durations are incompatible with complete colonic examination and most probably reflect sigmoidoscopies, incomplete procedures, or annotation error.

In a further analysis conducted at the level of the individual examination, each endoscopist’s mean inspection time, derived exclusively from their negative colonoscopies, in which no polyp was detected and no tissue was sampled, was assigned as an exposure to every procedure that endoscopist performed. The association of this assigned exposure with patient-level adenoma detection was then estimated by logistic regression with adjustment for age, sex, bowel-preparation quality, colonoscopy indication and institution. Because the exposure is constant within each endoscopist, all inference was based on cluster-robust standard errors with the endoscopist as the clustering unit, and the analysis was repeated within the screening population alone and within the combined screening and family-history population.

### 2.3. Statistical Analysis

The distribution of each continuous variable was examined graphically and by assessment of skewness and kurtosis. Normally distributed continuous variables are presented as mean ± standard deviation. Ordinal variables and continuous variables with non-normal distributions, including the BBPS score and withdrawal time, are presented as median with interquartile range (IQR), and categorical variables as frequencies and percentages. Because the BBPS distribution was bimodal, the complete frequency distribution of scores is also reported. Analyses were performed on complete cases. Withdrawal time was evaluated both as a continuous variable and in categorized form. Two categorization strategies were applied a priori: quartiles and clinically relevant intervals (<6, 6–8, 8–10 and >10 min). Additional analyses using finer intervals (0–4, 4–6, 6–8, 8–10, 10–15 and >15 min) were performed to characterize the shape of the association. The shape of the association between withdrawal time and adenoma detection was modeled flexibly using restricted cubic splines with five knots placed at the 5th, 27.5th, 50th, 72.5th and 95th percentiles of the withdrawal-time distribution, adjusted for age, sex and bowel-preparation quality. Non-linearity was assessed by a likelihood-ratio test against the corresponding linear model. Adjusted predicted probabilities were estimated across the range of withdrawal time by averaging over the observed distribution of covariates, and the marginal gain in adenoma detection per additional minute was derived as the first derivative of the fitted curve.

Non-normally distributed variables were compared across groups using the Kruskal–Wallis test and, for two-group comparisons, the Mann–Whitney U test. Associations between non-normally distributed variables were assessed using the Spearman rank correlation coefficient; the Pearson coefficient, including the point-biserial coefficient for binary outcomes, was reported in parallel. Trends in ADR and PDR across ordered withdrawal-time categories were assessed using the Cochran–Armitage test for trend.

Multivariable logistic regression was used to evaluate the independent association between withdrawal duration and adenoma detection, with polyp detection as a secondary outcome. Models were adjusted for age (continuous), sex, and bowel-preparation quality. Because the BBPS is an ordinal instrument with a non-linear distribution in this cohort, it was entered as a categorical covariate with a score of 6 as the reference category; models specifying BBPS as a continuous variable were also fitted and yielded materially identical estimates for withdrawal time. Results are reported as adjusted odds ratios (ORs) with 95% confidence intervals (CIs). Because procedures are clustered within endoscopists, all multivariable models were additionally fitted with cluster-robust Huber–White sandwich standard errors, using the endoscopist as the clustering unit. Two further specifications addressed clustering within institutions and within endoscopists. First, the principal model was refitted with fixed effects for institution. Second, fixed effects were added for the individual endoscopist, so that in this specification each procedure is compared only with other procedures performed by the same operator at the same institution; this approach absorbs all fixed differences between operators, including experience, technique and case mix, without requiring distributional assumptions about them.

The recorded withdrawal time incorporates time spent on biopsy, polypectomy and mucosal cleaning, and procedure-level associations with adenoma detection are therefore susceptible to reverse causation. Adenoma detection cannot be evaluated in procedures from which interventional time has been removed, because histological confirmation requires tissue sampling. We therefore performed an endoscopist-level analysis modeled on the design of Barclay et al. and on the definition of the withdrawal-time quality indicator adopted by the ASGE/ACG Task Force, in which average withdrawal time is measured in normal colonoscopies performed without biopsy sampling or polypectomy. For each endoscopist, inspection time was defined as the mean withdrawal time across colonoscopies in which no polyp was detected and no tissue was sampled, and each endoscopist’s ADR and PDR were calculated across all of their procedures. Analyses were restricted to endoscopists contributing at least 100 procedures and at least 30 negative examinations. Associations were quantified using Pearson and Spearman correlation coefficients and by linear regression of ADR on inspection time; detection rates were compared across quartiles of inspection time and above versus below both the historical 6 min benchmark and the 8 min minimum recommended in the 2024 ASGE/ACG quality indicators, using the Mann–Whitney U test.

Four pre-specified sensitivity analyses were performed. First, the primary multivariable model was refitted after excluding all procedures with a coded polypectomy. Second, the association between withdrawal duration and polyp detection was examined within the subgroup of procedures in which neither polypectomy nor biopsy was performed, in which the recorded withdrawal time contains no interventional component. Third, the endoscopist-level analysis was repeated after excluding endoscopists with a mean inspection time below 3 min, a duration incompatible with complete colonic examination. Fourth, the BBPS was modeled alternately as a continuous and a categorical covariate.

Receiver operating characteristic (ROC) analysis was used to assess the discriminatory performance of withdrawal duration for lesion detection and to identify the threshold maximizing the Youden index; this analysis was repeated within the subgroup of procedures performed without any intervention. Because the discriminatory capacity of withdrawal time was only moderate, ROC-derived thresholds are reported as descriptive summaries and are not proposed for individual case classification. Subgroup analyses were performed by sex and by age category (<50, 50–59, 60–69 and ≥70 years), and multiplicative interaction terms were fitted to assess effect modification. Subgroup and interaction analyses were exploratory, and no adjustment was made for multiple comparisons.

All tests were two-sided, and a *p*-value below 0.05 was considered statistically significant. Analyses were performed in Python version 3.12, using pandas version 3.0 for data management, NumPy version 2.4 and SciPy version 1.17 for numerical computation and hypothesis testing, scikit-learn version 1.8 for receiver operating characteristic analysis, and Matplotlib version 3.10 for figures. Logistic regression models were fitted by iteratively reweighted least squares, and cluster-robust standard errors were obtained from the Huber–White sandwich estimator with the endoscopist as the clustering unit. Restricted cubic spline bases were constructed using Harrell’s formulation. The analysis code is available from the corresponding author on reasonable request.

Because the recorded withdrawal time incorporates time spent on biopsy, polypectomy and mucosal cleaning, the analysis of inspection time at the level of the endoscopist, using withdrawal time measured exclusively in examinations in which no polyp was detected and no tissue was sampled, was regarded as the principal analysis addressing mucosal inspection effort. Procedure-level analyses describe the association with recorded withdrawal duration as captured in routine endoscopic practice and are presented as supportive and descriptive; they are not interpreted as estimates of net inspection time.

### 2.4. Ethics

The study was conducted in accordance with the principles of the Declaration of Helsinki and was approved by the Institutional Review Board (ASMC-0056-25) on 12 August 2025. Given the retrospective nature of the study and the use of de-identified data, the ethics committee waived informed consent.

## 3. Results

### 3.1. Withdrawal Duration and Lesion Detection Across the Cohort

A total of 31,780 colonoscopies performed between 1 January and 8 December 2025 were included in the final analysis. The median patient age was 60.4 years (IQR 53.5–68.3), 14,909 procedures (46.9%) were performed in male patients, and the median withdrawal time was 6.83 min (IQR 4.80–9.53). An indication could be assigned in 25,593 procedures (80.5%), of which screening was the most frequent (8229 procedures, 25.9%), followed by symptomatic or diagnostic presentation (7647, 24.1%), polyp surveillance (4142, 13.0%), family history of colorectal cancer (3672, 11.6%) and positive fecal occult blood testing (1903, 6.0%). Procedures were distributed across seven gastroenterology units, ranging from 1887 to 7646 examinations per unit, with the site not recorded for 263 procedures (0.8%). The median Boston Bowel-Preparation Scale score was 7 (IQR 6–9); the distribution was bimodal, with 15,709 procedures (49.4%) scored as 6 and 8052 (25.4%) scored as 9 (Table 1). Withdrawal time differed significantly across BBPS categories (Kruskal–Wallis H = 202.2; *p* < 0.001), although the magnitude of this association was negligible (Spearman ρ = −0.07).

**Figure 1:**
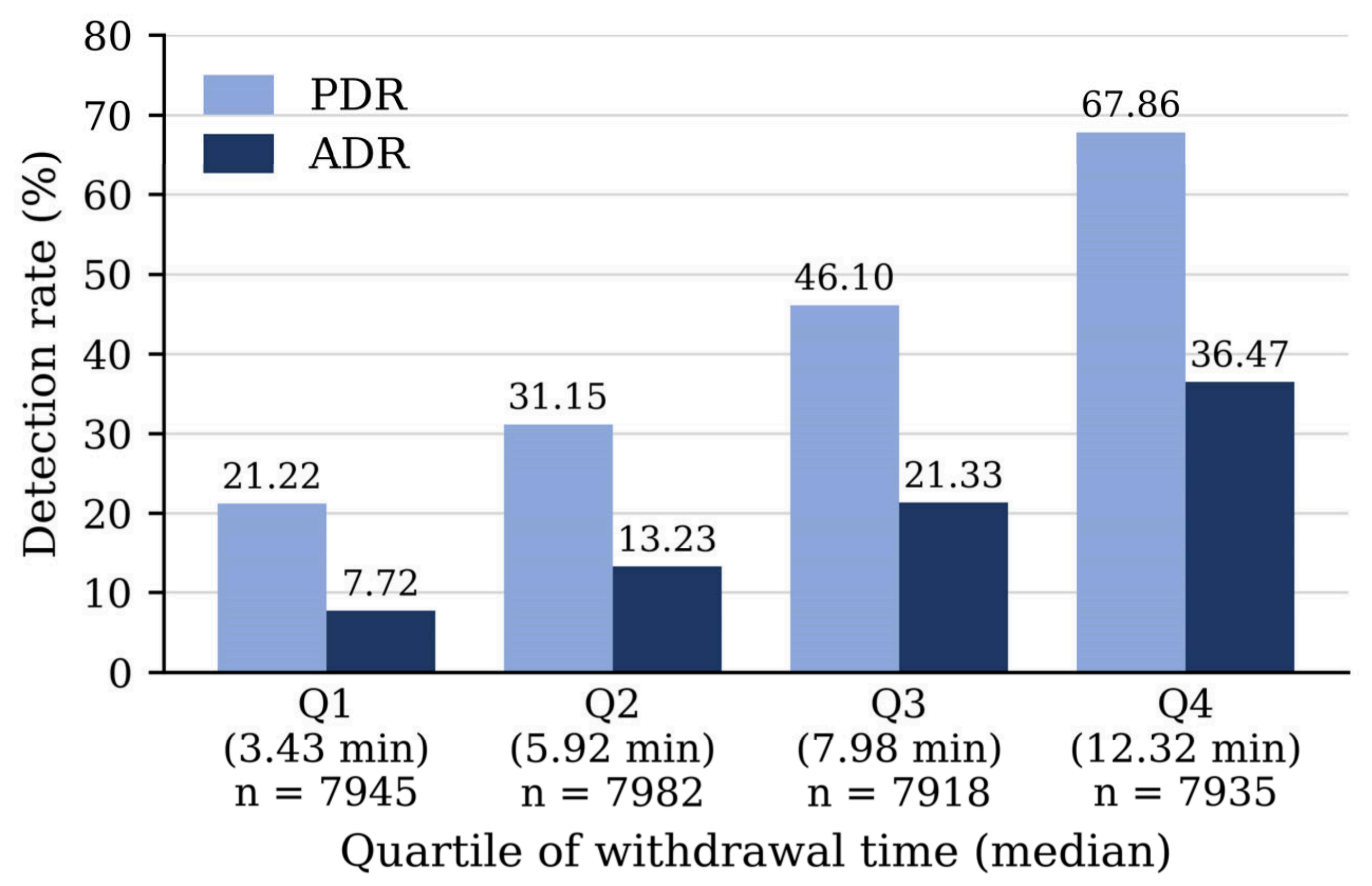
Adenoma and polyp detection according to quartile of colonoscopy withdrawal time. Bars show the proportion of examinations in which at least one adenoma (ADR) or at least one polyp (PDR) was detected, within quartiles of withdrawal time. Median withdrawal time and the number of procedures are shown beneath each quartile. ADR, adenoma detection rate; PDR, polyp detection rate.

Stratification according to withdrawal-time quartiles revealed a pronounced exposure–response gradient. Adenoma detection increased from 7.72% in the shortest quartile (median withdrawal time 3.43 min) to 36.47% in the longest quartile (median withdrawal time 12.32 min), representing an approximately 4.7-fold difference in detection. Polyp detection increased in parallel across the same quartiles, from 21.22% to 67.86% (Figure 1). The association followed a monotonic pattern across the intermediate quartiles rather than a binary threshold transition, supporting a graded relationship between withdrawal duration and neoplasia detection.

The same gradient was evident when procedures were grouped into clinically relevant withdrawal intervals (Table 2). Adenoma detection rose from 9.30% in examinations completed in under six minutes to 16.79%, 24.60% and 37.64% across successive categories, while polyp detection rose from 23.93% to 37.79%, 52.75% and 68.94%. Examinations exceeding ten minutes were associated with a more than fourfold higher ADR than those completed below the historical six-minute benchmark. Formal trend testing confirmed highly significant ordered increases in both outcomes across progressively longer withdrawal categories (Cochran–Armitage χ^2^ for trend 2354.12 for adenoma detection and 4035.04 for polyp detection; both *p* < 0.001). Collectively, these findings indicate that the relationship between withdrawal duration and lesion detection extends beyond adherence to a minimum procedural standard and instead reflects a continuous gradient in the thoroughness of mucosal inspection. These procedure-level figures describe the association with recorded withdrawal duration, which includes time spent on biopsy, polypectomy and mucosal cleaning. The principal analysis of inspection effort, conducted at the level of the endoscopist in examinations containing no intervention, is presented below.

### 3.2. Supportive Analysis: Recorded Withdrawal Duration and Adenoma Detection at the Procedure Level

To determine whether the association between withdrawal duration and adenoma detection persisted after adjustment for baseline demographic and procedural factors, multivariable logistic regression analyses were performed (Table 3). Each additional minute of withdrawal time was associated with a 14% increase in the odds of adenoma detection (adjusted OR 1.14, 95% CI 1.13–1.15; *p* < 0.001) after adjustment for age, sex and bowel-preparation quality. Categorical modeling demonstrated a progressive increase in detection probability with longer withdrawal: relative to procedures completed in under 6 min, the adjusted odds of adenoma detection were 1.93 (95% CI 1.77–2.10) for withdrawal times of 6–8 min, 3.07 (95% CI 2.80–3.36) for 8–10 min and 5.54 (95% CI 5.12–5.99) for durations exceeding 10 min (all *p* < 0.001). The graded increase in adjusted odds across categories is consistent with a dose-dependent relationship rather than a simple threshold effect.

To express this association on an absolute scale, model-predicted adenoma detection was estimated at fixed withdrawal times with all covariates held at their observed values. Predicted detection rose from 13.6% at six minutes to 21.0% at eight minutes and 28.2% at ten minutes, corresponding to approximately seven and fifteen additional adenoma-positive examinations per hundred procedures.

Because colonoscopies performed by the same endoscopist are not statistically independent, all models were refitted using cluster-robust standard errors with the endoscopist as the clustering unit. Point estimates were unchanged, and confidence intervals widened only modestly (Table 3), indicating that the precision of the procedure-level model was not materially overstated. As expected from the established epidemiology of colorectal neoplasia, older age and male sex were independently associated with higher adenoma detection; these associations were consistent with previous reports and are presented in full in Supplementary Table S1.

Because case mix, the institution and the individual endoscopist are plausible sources of confounding, models with progressive adjustment for these factors were fitted, together with analyses restricted to homogeneous subgroups; these are reported with the other sensitivity analyses below. Estimates obtained under alternative specifications of clustering, including cluster-robust standard errors with the endoscopist as the clustering unit, are presented in Supplementary Table S2.

#### 3.2.1. Withdrawal Duration Is Correlated with Both Polyp and Adenoma Detection

Correlation analyses were performed to characterize the association between withdrawal duration and lesion detection as continuous relationships (Table 4). Withdrawal time was significantly and positively correlated with both polyp detection (Pearson r = 0.346; Spearman ρ = 0.364) and adenoma detection (r = 0.279; ρ = 0.276), with all *p* values below 0.001. The consistency of the Pearson and Spearman estimates indicates that these associations are not driven by extreme values of withdrawal time.

The coefficient for polyp detection exceeded that for adenoma detection, which is expected given that adenomas constitute a subset of detected polyps and that the two outcomes differ substantially in base rate. These coefficients should therefore be interpreted as confirming the direction and consistency of the association rather than as a comparison of the relative sensitivity of withdrawal time to different lesion types. The number of polypectomies recorded within the procedure annotation was not analyzed, as this field was populated in only a small minority of examinations and does not constitute a valid measure of polyp burden.

#### 3.2.2. Operating Characteristics of Withdrawal Time for Adenoma Detection

**Figure 2:**
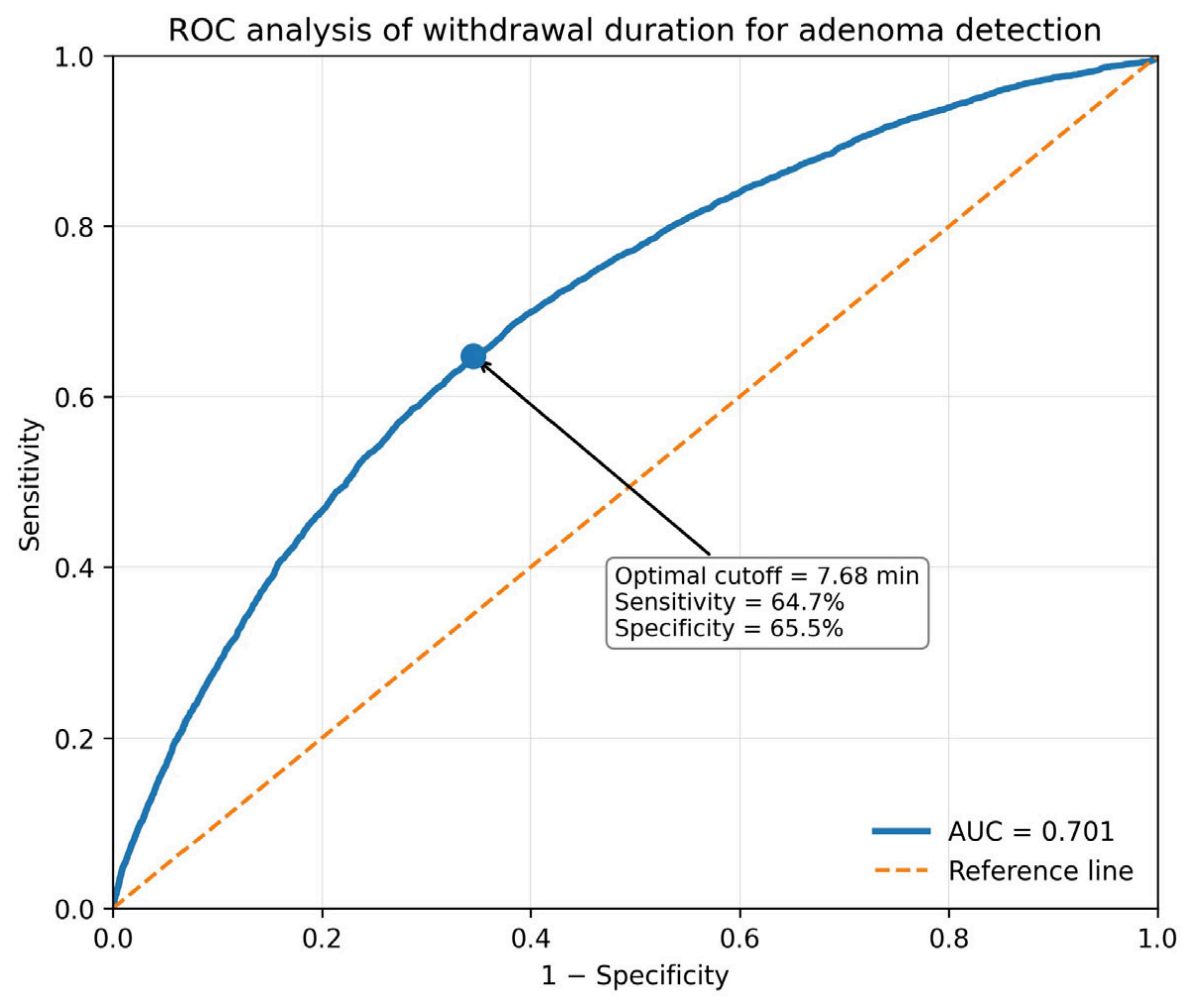
ROC-derived performance of withdrawal duration for adenoma detection. Withdrawal time demonstrated moderate discrimination for ADR prediction (AUC 0.701), with a Youden-optimal region of approximately 7 to 8 min.

Receiver operating characteristic analysis was performed to characterize the discriminatory performance of withdrawal duration for adenoma detection. Withdrawal time demonstrated moderate discrimination, with an area under the curve of 0.701 (Figure 2). Because the area under the curve is a global property of the curve and is not specific to any single threshold, the operating characteristics of withdrawal time were additionally examined across a range of clinically relevant cut-offs (Table 5).

Positive predictive value increased progressively as the threshold was raised, from 26.2% at 6 min to 29.4% at 7 min, 32.3% at 8 min and 37.6% at 10 min, while sensitivity fell correspondingly from 81.9% to 71.7%, 61.4% and 42.4%. This pattern accounts for the higher detection rates observed in the longer withdrawal-time categories and reflects progressive enrichment of the group examined rather than improved discrimination. The Youden index was relatively flat across the intermediate range, with values of 0.295 at 7 min, 0.302 at 7.68 min, and 0.299 at 8 min, indicating that no single threshold was clearly superior and that the Youden-optimal region lies at approximately 7 to 8 min. When the analysis was restricted to the 15,487 procedures performed without polypectomy or biopsy, the area under the curve was 0.657, and the Youden-optimal threshold was 6.43 min.

### 3.3. The Effect of Withdrawal Duration Persists Across Demographic Risk Strata

**Figure 3:**
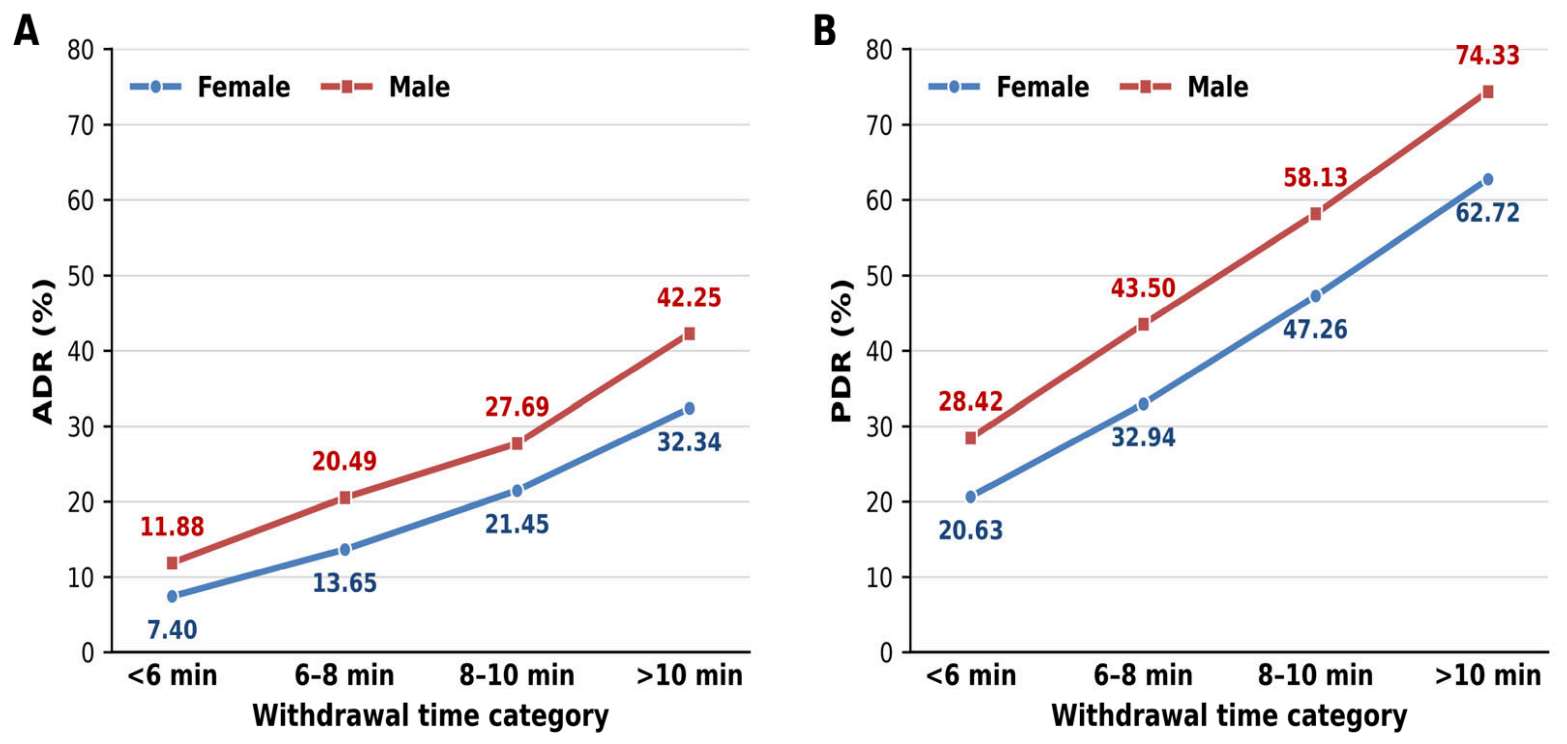
Adenoma and polyp detection rates by colonoscopy withdrawal time category, stratified by sex. (**A**) Adenoma detection rate (ADR, %). (**B**) Polyp detection rate (PDR, %). Rates rose monotonically across withdrawal time categories in both sexes and were consistently higher in males.

Because the prevalence of colorectal neoplasia varies by sex, analyses were stratified accordingly (Table 6, Figure 3). Adenoma and polyp detection were consistently higher in men than in women within every withdrawal-time category, as expected from the established epidemiology of colorectal neoplasia. The exposure–response gradient was nonetheless preserved in both sexes.

Among women, adenoma detection increased from 7.40% in examinations completed in under six minutes to 13.65%, 21.45%, and 32.34% across the 6–8, 8–10, and greater than 10 min categories (Figure 3A); polyp detection increased from 20.63% to 62.72% across the same categories (Figure 3B). Among men, adenoma detection increased from 11.88% to 20.49%, 27.69%, and 42.25% (Figure 3A), and polyp detection from 28.42% to 74.33% (Figure 3B). Tests for trend were highly significant in both sexes for both outcomes (all *p* < 0.001). The relative increase across categories was of similar magnitude in women and men, indicating that the association between prolonged withdrawal and lesion detection is preserved across sex strata despite the difference in baseline neoplasia prevalence.

**Figure 4:**
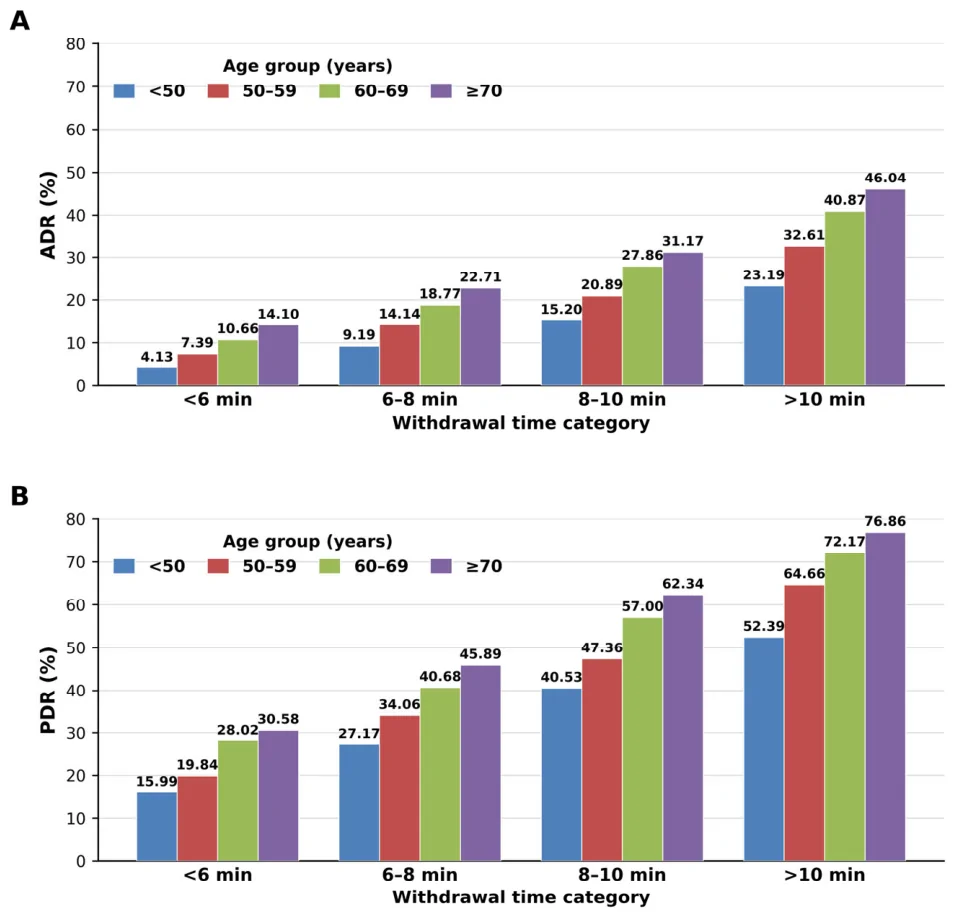
Adenoma and polyp detection rates by colonoscopy withdrawal time category, stratified by patient age group. (**A**) Adenoma detection rate (ADR, %). (**B**) Polyp detection rate (PDR, %). Within every withdrawal time category, detection rose stepwise across age groups, and within every age group it rose stepwise with longer withdrawal time; the highest rates occurred in patients aged ≥70 years examined for >10 min.

Age-stratified analyses demonstrated a persistent exposure–response association between withdrawal duration and lesion detection in every age group (Figure 4; Table 7). Although baseline adenoma prevalence rose with age across all withdrawal categories, progressively longer withdrawal was consistently associated with higher detection within each stratum. Among patients younger than 50 years, adenoma detection increased from 4.13% in examinations completed in under six minutes to 9.19%, 15.20% and 23.19% across the 6–8, 8–10 and greater than 10 min categories. Comparable gradients were observed in patients aged 50–59 years (7.39% to 32.61%), 60–69 years (10.66% to 40.87%) and 70 years or older (14.10% to 46.04%). Parallel increases were seen for polyp detection in every stratum, and tests for trend were significant throughout (all *p* < 0.001).

Notably, while the relative gradient was similar across age groups, the absolute gain associated with longer withdrawal widened with increasing age: the difference in adenoma detection between the shortest and longest withdrawal categories rose from 19.1 percentage points in patients under 50 years to 31.9 percentage points in those aged 70 years or older. These findings indicate that the association between prolonged withdrawal and lesion detection is preserved across the age range, and that the absolute diagnostic yield of unhurried inspection is greatest in older patients.

No interaction was observed between age and withdrawal duration in their association with adenoma detection (interaction odds ratio 1.000, 95% CI 0.999–1.001 per year-minute; equivalent to 0.999, 95% CI 0.992–1.006 per decade of age; likelihood-ratio χ^2^ = 0.062, one degree of freedom, *p* = 0.80). Estimates for withdrawal time, age, sex and bowel-preparation quality were essentially unchanged from the main model, and confidence intervals widened slightly with the inclusion of the interaction term, as expected.

Stratified estimates were consistent with the absence of interaction: the adjusted odds ratio per additional minute was 1.148 (95% CI 1.121–1.176) in patients younger than 50 years, 1.139 (1.127–1.151) at 50 to 59 years, 1.144 (1.132–1.156) at 60 to 69 years and 1.136 (1.123–1.150) at 70 years or older, with corresponding values of 1.147 (1.137–1.157) in women and 1.135 (1.126–1.145) in men (Supplementary Table S3).

The absence of multiplicative interaction indicates that the relative association with withdrawal duration was similar across the age range. It does not imply a constant absolute difference, as shown in Table 7; the absolute difference in adenoma detection between the shortest and longest withdrawal categories increased from 19.1 percentage points in patients younger than 50 years to 31.9 percentage points in those aged 70 years or older.

#### 3.3.1. Non-Linear Modeling of the Withdrawal Time–Detection Relationship

To characterize the shape of the relationship between withdrawal duration and lesion detection more finely, detection rates were examined across six withdrawal-time intervals (Table 8). Adenoma detection increased monotonically from 6.90% in examinations completed within four minutes to 11.21%, 16.79%, 24.60%, 34.24% and 45.38% across successive intervals, and polyp detection rose in parallel from 19.47% to 78.40%. Tests for trend were highly significant for both outcomes (both *p* < 0.001).

**Figure 5:**
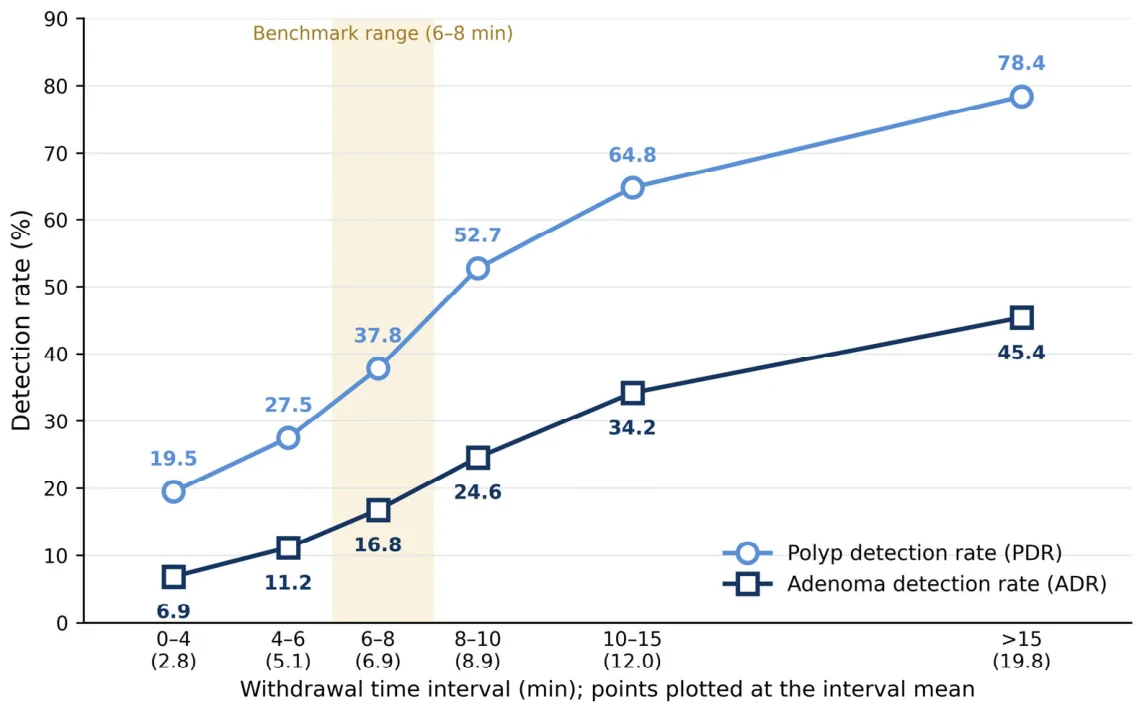
Adenoma and polyp detection across finely stratified withdrawal-time intervals. Detection rates increased across progressively longer intervals at the procedure level. Recorded withdrawal times include time spent on biopsy and polypectomy.

At the procedure level, no plateau was evident within the intervals examined, and detection continued to rise across the longest withdrawal durations (Figure 5). This observation requires two qualifications. First, the recorded withdrawal time includes time spent on biopsy, polypectomy, and mucosal cleaning, so examinations containing multiple lesions are necessarily assigned to longer intervals; the continued rise therefore reflects, in part, the interventional component of the recorded duration rather than inspection effort alone. Second, when the analysis was repeated at the level of the endoscopist using inspection time measured in examinations containing no intervention, the gradient attenuated beyond approximately seven minutes. These findings should accordingly be interpreted as associations rather than as evidence of a causal effect of prolonged inspection at every duration.

**Figure 6:**
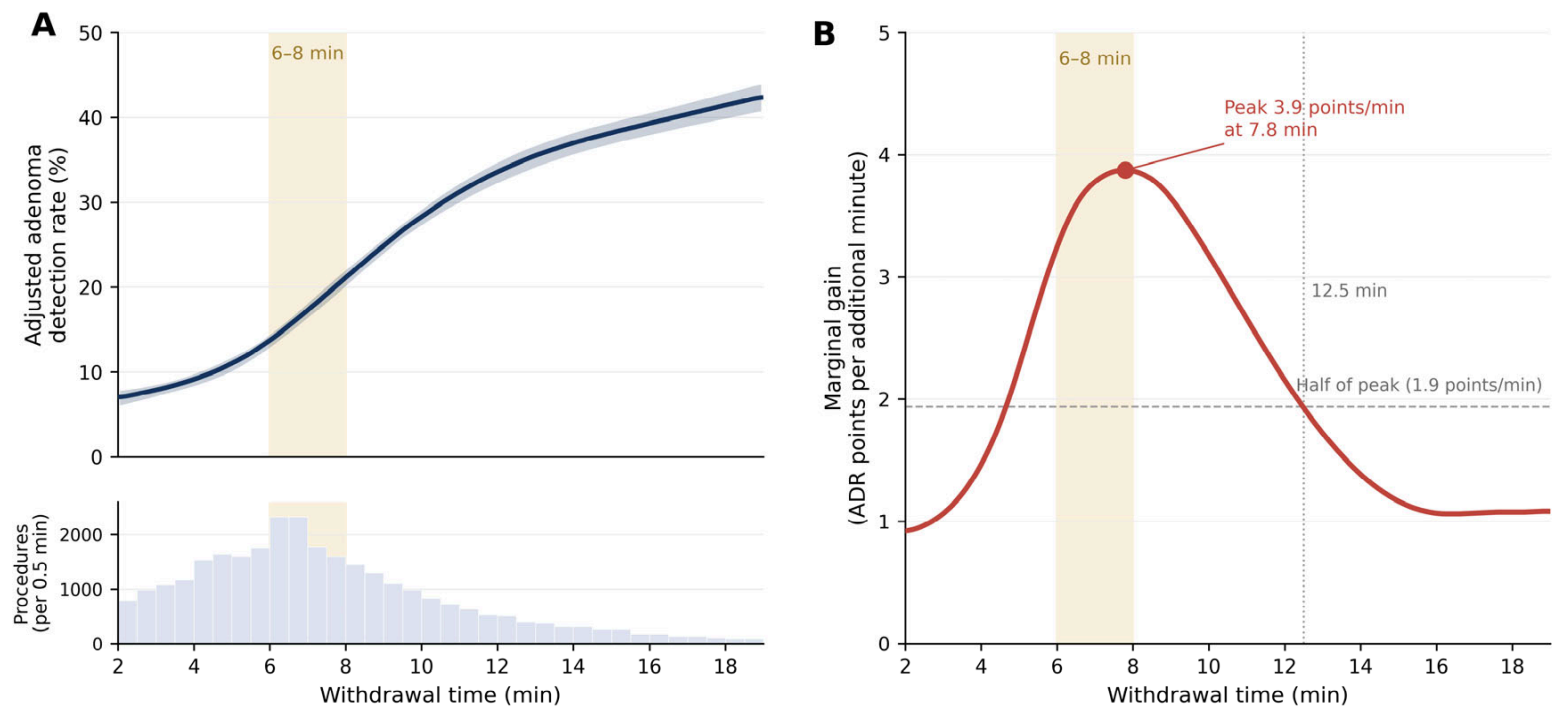
Restricted cubic spline model of adjusted adenoma detection rate across withdrawal time. (**A**) Adjusted ADR (%) as a continuous function of withdrawal time (restricted cubic spline, 5 knots; shaded band, 95% CI); the lower strip shows the distribution of procedures across withdrawal time (per 0.5-min interval). (**B**) Marginal gain, i.e. the additional ADR points obtained per additional minute of withdrawal, derived from the first derivative of the spline in (**A**). Marginal gain peaked at 3.9 ADR points/min at 7.8 min and fell below half of peak beyond 12.5 min. Shading marks the 6–8 min benchmark range.

To model the shape of the association without imposing categorical boundaries, withdrawal time was fitted as a restricted cubic spline. Departure from linearity was highly significant (likelihood-ratio χ^2^ = 337.8, three degrees of freedom; *p* < 0.001). Adjusted adenoma detection rose from 9.0% at four minutes to 13.6% at six, 21.0% at eight, 28.2% at ten and 33.5% at twelve minutes, reaching 41.4% at eighteen minutes (Figure 6A). The marginal gain per additional minute was not constant: it increased from 1.5 percentage points at four minutes to a maximum of 3.9 percentage points at 7.8 min, then declined progressively to 2.1 points at twelve minutes and 1.2 points at fifteen minutes (Figure 6B). Expressed as two-minute increments, adenoma detection increased by 7.4 percentage points between six and eight minutes and 7.2 points between eight and ten minutes, but by only 3.4 points between twelve and fourteen minutes and 2.3 points between fourteen and sixteen minutes. The flexible model therefore demonstrates a plateau that the categorical analysis did not resolve, with the point of maximal marginal return occurring at approximately eight minutes.

#### 3.3.2. Principal Analysis: Inspection Time Measured at the Endoscopist Level in Negative Examinations

Adenoma detection could not be evaluated after excluding interventional procedures, since all 6252 adenomas (100%) occurred in the 16,293 procedures (51.3%) in which tissue was sampled. We therefore examined the association at the endoscopist level. Inspection time was calculated from 14,611 colonoscopies in which no polyp was detected and no tissue was sampled. After restriction to endoscopists with at least 100 procedures and at least 30 negative examinations, 107 endoscopists contributing 26,793 procedures were analyzed. Median inspection time was 6.6 min (IQR 4.9–7.7; range 2.0–13.3), and median endoscopist ADR was 18.8% (range 5.3–41.3%), indicating substantial between-operator variation.

Inspection time correlated significantly with adenoma detection (Spearman ρ = 0.46, *p* < 0.001) and with polyp detection (ρ = 0.33, *p* < 0.001). ADR increased across quartiles of inspection time from 14.6% to 21.0%, 22.2% and 23.6% (Table 9), corresponding to an absolute increase of approximately 1.5 percentage points per additional minute of inspection. Endoscopists achieving a mean inspection time of at least 6 min, the historical benchmark, had a higher ADR than those below it (21.8% vs. 15.7%, *p* < 0.001), and the same was true of the 8 min minimum recommended in the 2024 ASGE/ACG quality indicators (22.3% vs. 18.6%, *p* = 0.02). Sixty-four of the 107 endoscopists (59.8%) met the historical 6 min benchmark, whereas only 20 (18.7%) met the current 8 min recommendation. Three endoscopists (471 procedures, 1.8%) had a mean inspection time below 3 min; their exclusion did not materially alter the association (ρ = 0.42, *p* < 0.001; Table 10). The increase in ADR was most pronounced between the first and second quartiles and became more gradual thereafter, indicating attenuation of the gradient once inspection times exceeded approximately six to seven minutes.

Two additional procedure-level sensitivity analyses were consistent with these findings. After exclusion of all procedures with a coded polypectomy (n = 22,997; 1835 adenomas; ADR 8.0%), each additional minute of withdrawal time remained associated with adenoma detection (adjusted OR 1.19, 95% CI 1.18–1.20; *p* < 0.001). Among the 15,487 procedures in which no polypectomy or biopsy was performed, PDR increased from 3.4% in procedures shorter than 6 min to 5.1%, 8.9% and 13.5% across successive withdrawal categories (*p* for trend < 0.001), with an adjusted OR of 1.14 (95% CI 1.12–1.15) per additional minute (Table 11 and Table 12). The gradient was preserved within every sex and age stratum of this subgroup, with significant trends across withdrawal categories throughout (Supplementary Table S4). In this intervention-free subgroup, the ROC-derived threshold was 6.43 min (AUC 0.657), lower than the 7.68 min observed in the full cohort, consistent with partial inflation of the latter by interventional time. Taken together, six analyses using different designs and analytic samples yielded concordant estimates, indicating that the association is not attributable to the inclusion of interventional time within the recorded withdrawal duration (Supplementary Table S5).

To relate inspection effort directly to patient-level outcomes, each endoscopist’s mean inspection time, derived exclusively from their negative examinations, in which no polyp was detected and no tissue was sampled, was assigned as an exposure to every procedure that endoscopist performed (26,793 procedures; adenoma detection 20.05%). Across quartiles of this assigned exposure, adenoma detection rose from 14.21% among procedures performed by endoscopists in the lowest quartile (mean inspection time 3.62 min) to 20.90% and 23.03% in the second and third quartiles (5.56 and 6.86 min) and was 22.12% in the highest quartile (8.59 min), again indicating attenuation of the gradient at longer inspection times. In logistic regression adjusted for age, sex and bowel-preparation quality, each additional minute of assigned inspection time was associated with adenoma detection (adjusted OR 1.086, 95% CI 1.046–1.129; *p* < 0.001, cluster-robust). The estimate was materially unchanged after further adjustment for colonoscopy indication (1.090, 95% CI 1.045–1.136) and for indication and institution together (1.086, 95% CI 1.046–1.129). Within the screening population alone (n = 6824) the association was somewhat stronger (adjusted OR 1.121, 95% CI 1.068–1.177), and within the combined screening and family-history population (n = 9947) it was 1.098 (95% CI 1.055–1.142) (Supplementary Table S6). On the absolute scale, predicted adenoma detection rose from 18.5% at an endoscopist mean inspection time of five minutes to 22.3% at eight minutes, a difference of 3.8 percentage points.

Because the recorded withdrawal time in procedures without a confirmed cecal landmark defaulted to approximately the entire procedure duration, all analyses were repeated with the cohort restricted to the 29,607 procedures in which the landmark was confirmed. Within this restricted cohort, inspection time could be derived from 13,500 negative examinations for 101 endoscopists contributing 25,003 procedures, and inspection time remained associated with adenoma detection at the endoscopist level (Spearman ρ = 0.456; *p* < 0.001). In the assigned-exposure model adjusted for age, sex, bowel-preparation quality, colonoscopy indication and institution, each additional minute of inspection time was associated with adenoma detection (adjusted OR 1.091, 95% CI 1.050–1.134; *p* < 0.001, cluster-robust), and within the screening population alone (n = 6400) the estimate was 1.118 (95% CI 1.060–1.180). The supportive procedure-level estimate was 1.175 (95% CI 1.152–1.198) per additional minute, compared with 1.141 (95% CI 1.125–1.157) in the full cohort, the difference being consistent with removal of the inflated withdrawal times described above (Supplementary Table S7).

**Figure 7:**
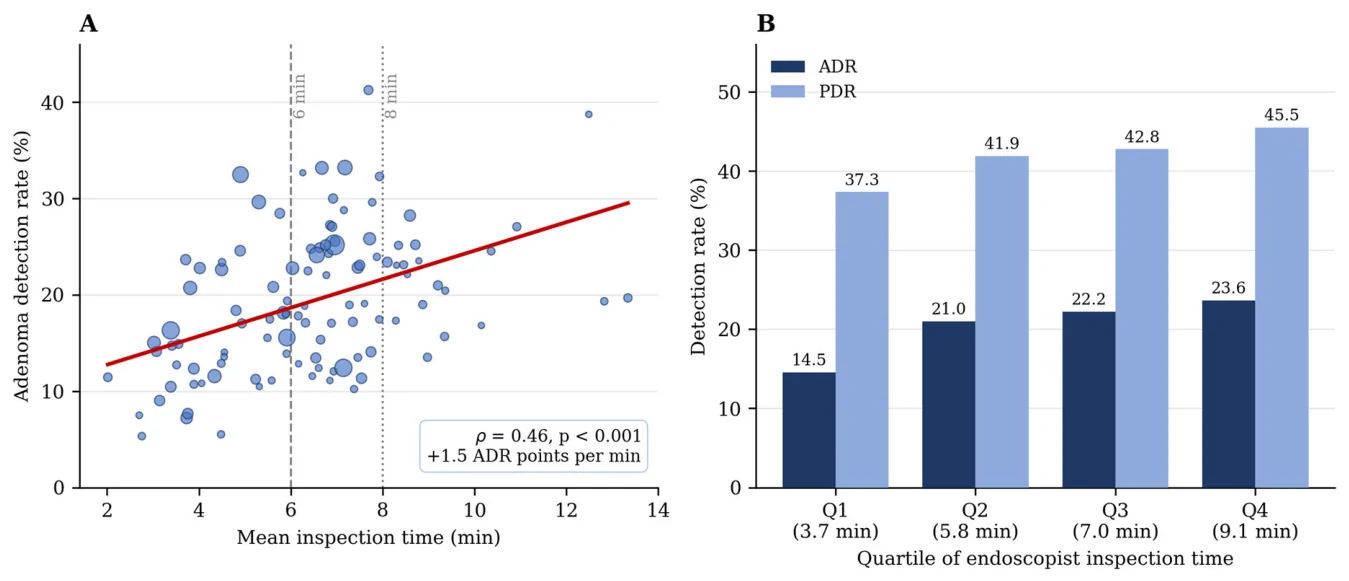
Endoscopist-level association between inspection time and lesion detection. (**A**) Adenoma detection rate plotted against mean inspection time for each of the 107 endoscopists contributing ≥100 procedures and ≥30 negative examinations; symbol size is proportional to procedure volume, and the line represents the fitted linear regression (Spearman ρ = 0.46, *p* < 0.001; +1.5 percentage points of ADR per additional minute). Dashed and dotted lines mark the 6 min and 8 min quality benchmarks. (**B**) Adenoma and polyp detection rates across quartiles of endoscopist inspection time, weighted by procedure volume. Inspection time was measured exclusively in colonoscopies in which no polyp was detected and no tissue was sampled.

The relationship between endoscopist inspection time and lesion detection is displayed in Figure 7. Detection increased steeply across the lower range of inspection times and more gradually beyond approximately seven minutes, with substantial residual variation between endoscopists at any given inspection time, indicating that inspection duration is an important but not exclusive determinant of detection performance.

#### 3.3.3. Sensitivity Analyses Addressing Confounding and Exposure Measurement

Because indications for colonoscopy differ in both baseline lesion prevalence and interventional burden, the observed association could in principle reflect case mix rather than inspection effort. Adenoma detection ranged from 15.29% among symptomatic examinations to 38.26% following a positive fecal occult blood test, and median withdrawal time varied correspondingly, from 6.48 min at screening to 8.10 min in the fecal occult blood group (Table 13). The magnitude of this heterogeneity confirms that indication is a plausible confounder.

To determine whether the association was attributable to case mix or to differences between operators and institutions, models were fitted with progressive adjustment (Table 14). Adjustment for indication gave an adjusted odds ratio of 1.135 (95% CI 1.128–1.143) per additional minute, and further adjustment for institution gave 1.142 (1.135–1.150). With fixed effects for the individual endoscopist added, so that each procedure was compared only with other procedures performed by the same operator at the same institution, the estimate rose to 1.183 (1.173–1.192). Adenoma detection varied substantially across institutions, from 14.47% to 27.03%, and across endoscopists, from 5.3% to 41.3%. Refitting the principal model with cluster-robust standard errors clustered on the endoscopist left the point estimate unchanged (1.141, 95% CI 1.125–1.157). The association therefore did not attenuate under adjustment for case mix, institution or operator, and was somewhat stronger when the endoscopist was held constant.

A pre-specified analysis was restricted to the 8229 examinations performed for screening, in which baseline lesion prevalence is most homogeneous. Within this population, the adjusted odds ratio was 1.159 (95% CI 1.143–1.174) per additional minute, and adenoma detection rose from 7.22% in examinations completed in under six minutes to 13.67%, 20.61% and 32.60% across successive withdrawal categories. Restricting instead to screening and family history combined (n = 11,901) gave 1.148 (1.136–1.161). The estimates obtained within these restricted populations were larger than those obtained in the full cohort, which is the opposite of the attenuation that case-mix confounding would produce.

Withdrawal time was derived from automated annotation of the cecal and rectal landmarks, and a cecal landmark was confirmed in 29,607 procedures (93.2%). In a sensitivity analysis restricted to these procedures, the association was preserved and somewhat stronger than in the full cohort (adjusted odds ratio 1.175, 95% CI 1.166–1.184 per additional minute), with adenoma detection rising from 9.14% in examinations completed in under six minutes to 16.98%, 25.53% and 40.76% across successive categories. Procedures without a confirmed cecal landmark had a longer median withdrawal time than those with one (12.25 versus 6.62 min). This difference was attributable to the withdrawal interval defaulting to approximately the entire procedure when the landmark was not identified: the ratio of withdrawal time to total procedure duration was 1.000 in these examinations compared with 0.520 in those with a confirmed landmark, while total procedure duration was indistinguishable between the two groups (median 13.2 min in both). These procedures therefore reflect annotation failure with an overestimated withdrawal time rather than incomplete or abbreviated examinations.

Finally, an exploratory analysis examined advanced histology, defined as tubulovillous or villous architecture, dysplasia within an adenoma, or invasive carcinoma. Advanced histology was identified in 514 procedures, representing 1.62% of the cohort and 8.2% of all adenomas, and its detection increased across withdrawal-time categories from 0.56% to 0.93%, 1.39% and 4.37% (adjusted odds ratio 1.127, 95% CI 1.114–1.140 per additional minute; Table 15). This analysis is subject to the same limitation as the primary outcome, and arguably to a greater degree, since larger and more complex lesions require additional resection time. Sessile serrated lesions could not be evaluated, as no entry carrying a serrated descriptor was present in the histopathology coding for the study period.

Taken together, these analyses demonstrate a consistent association between prolonged withdrawal examination and lesion detection that persisted after adjustment for demographic factors, colonoscopy indication, institution and individual endoscopist, across sex and age strata, within a homogeneous screening population, and when inspection time was measured at the level of the endoscopist in examinations containing no intervention. The gradient was steepest across shorter withdrawal durations and attenuated beyond approximately seven to eight minutes, indicating that withdrawal time operates as a graded rather than a dichotomous marker of inspection quality, with diminishing incremental returns at longer durations.

## 4. Discussion

In this multicenter cohort of 31,780 colonoscopies performed across seven endoscopy units, withdrawal duration was independently associated with adenoma detection. Adenoma detection increased progressively across withdrawal-time categories rather than showing a simple threshold effect at the historical six-minute benchmark, and each additional minute of withdrawal was associated with higher odds of adenoma detection after adjustment for age, sex, and bowel-preparation quality. Flexible modeling showed that the association was markedly non-linear, with the marginal gain in detection per additional minute reaching a maximum at approximately eight minutes and diminishing thereafter. Because recorded withdrawal times incorporate the time required for biopsy and polypectomy, we additionally examined the association at the level of the individual endoscopist, using inspection time measured exclusively in examinations in which no polyp was detected, and no tissue was sampled. Among 107 endoscopists contributing 26,793 procedures, inspection time correlated with adenoma detection (Spearman ρ = 0.46; *p* < 0.001), confirming that the association is not an artifact of interventional time. The association was consistent across age and sex strata and was robust to adjustment for clustering within endoscopists. Because the recorded withdrawal time incorporates interventional time, the procedure-level estimates describe the association with total recorded duration rather than with mucosal inspection effort. The analysis conducted at the level of the endoscopist, in which inspection time was measured only in examinations containing no intervention, is therefore the principal estimate of the relationship between inspection effort and detection, while the procedure-level findings characterize the association as it appears in routinely recorded data. When each endoscopist’s mean inspection time was assigned to their individual procedures and related to patient-level adenoma detection with adjustment for age, sex, bowel-preparation quality, colonoscopy indication and institution, the association persisted (adjusted odds ratio 1.086 per additional minute, 95% CI 1.046–1.129), and was somewhat stronger within the screening population (1.121, 95% CI 1.068–1.177).

The relationship between withdrawal duration and adenoma detection has been recognized for nearly two decades [16,20,21,22]. Landmark work by Barclay and colleagues showed that endoscopists with mean withdrawal times of at least six minutes achieved higher ADR than those with shorter withdrawal times, leading to the incorporation of the six-minute benchmark into colonoscopy quality standards [16]. Subsequent studies and meta-analyses have generally supported the association between longer withdrawal time and improved lesion detection [23,24]. However, much of the existing literature has compared performance above and below predefined cut-offs, leaving uncertainty regarding the shape of the association beyond the minimum recommended withdrawal time [25], and relatively few studies have separated the time spent inspecting the mucosa from the time spent performing therapeutic maneuvers.

Our findings suggest that withdrawal time should be viewed not only as a minimum quality requirement but also as a graded marker of careful mucosal inspection, which has direct implications for clinical practice. ADR and PDR increased across progressively longer withdrawal intervals, supporting the concept that more meticulous inspection is associated with improved diagnostic yield. Longer withdrawal may allow better visualization behind folds, more careful cleaning of residual debris, repeated assessment of difficult segments, and improved recognition of subtle lesions, including flat adenomas and sessile serrated lesions [17,25].

Our endoscopist-level findings correspond closely to those of the foundational study in this field. Barclay and colleagues, examining twelve gastroenterologists in a community practice, reported that withdrawal times in procedures without polypectomy ranged from 3.1 to 16.8 min and that adenoma detection ranged from 9.4% to 32.7% across operators; those achieving a mean of at least six minutes detected neoplasia in 28.3% of examinations compared with 11.8% among faster operators [16]. Our analysis, conducted in 107 endoscopists rather than twelve, reproduced both features: inspection time ranged from 2.0 to 13.3 min, adenoma detection ranged from 5.3% to 41.3%, and endoscopists meeting the then-current six-minute benchmark achieved a higher detection rate than those below it (21.8% versus 15.7%). The absolute difference in our cohort was smaller than that reported by Barclay, which is expected given that our cohort comprised screening, surveillance and diagnostic examinations rather than screening alone, and that overall practice standards have risen since 2006. The persistence of a comparable between-operator distribution across two decades and two health systems is nonetheless notable, and the wide variation we observed indicates that inspection behavior remains a substantial and modifiable source of heterogeneity in colonoscopy quality. Consistent with this, the association between withdrawal duration and adenoma detection was not attenuated when fixed effects for institution and for the individual endoscopist were included in the procedure-level model; the estimate rose from 1.14 to 1.18 per additional minute, indicating that differences between operators in experience, technique or case mix do not account for the relationship.

The magnitude and shape of the association also merit direct comparison with previous reports. Butterly and colleagues, analyzing 7996 colonoscopies performed by 42 endoscopists in the New Hampshire Colonoscopy Registry, found that polyp and adenoma detection were highest among endoscopists with a median normal withdrawal time of nine minutes, with detection of clinically significant serrated polyps peaking at eight to nine minutes [25]; in our cohort the highest quartile of inspection time, with a mean of 9.1 min, likewise showed the highest detection rates. Desai and colleagues, in a prospective multicenter randomized trial in which withdrawal time was recorded with resection and cleaning time excluded, reported a 6% increase in the odds of adenoma detection per additional minute, with detection rising up to approximately thirteen minutes [26]. Our procedure-level estimate was larger, at 14% per minute, which is consistent with the residual contribution of interventional time to the recorded withdrawal duration in our dataset; differences in case mix are also relevant, since their trial population had ADR of 49.6% and 63.9% in the screening and surveillance groups respectively, compared with 19.7% in our unselected cohort. Randomized evidence supports the gradient we describe: Zhao and colleagues randomized 1027 patients to a minimum withdrawal of nine or six minutes and reported higher adenoma detection in the nine-minute group (36.6% versus 27.1%) together with higher polyp detection (58.0% versus 47.8%) [27], a subsequent tandem trial demonstrated a substantially lower adenoma miss rate with the longer withdrawal [28], and a meta-analysis of three randomized trials estimated a 23% relative increase in adenoma detection with a nine-minute withdrawal [24]. Our observational estimates cannot establish causality, but their direction and magnitude are concordant with these trial data.

Taghiakbari and colleagues addressed the same measurement problem directly, deriving a corrected withdrawal time from full-length video recordings in which the duration of interventions is subtracted to approximate mucosal inspection time [23]. Among 1072 colonoscopies performed by 15 endoscopists, they reported a 7.1% increase in the odds of adenoma detection per corrected minute, with absolute gains of 1.1 to 1.3 percentage points per minute among high-performing endoscopists and 1.2 to 3.4 points among low performers and estimated that low performers required approximately eleven minutes of corrected withdrawal time to reach an ADR of 26%. Our endoscopist-level estimate of approximately 1.5 percentage points per additional minute of inspection time falls within this range, despite a different method of deriving inspection time and a cohort thirty times larger. Their observation that the benefit was greatest among low performers, and their consequent argument for individualized rather than universal withdrawal-time targets, is consistent with the wide residual variation we observed between endoscopists at any given inspection time.

The shape of the association was characterized using a flexible model rather than categorical intervals. Adjusted adenoma detection rose steeply between approximately five and ten minutes and then progressively flattened, with the marginal gain per additional minute peaking at 3.9 percentage points at 7.8 min and falling below half of that value beyond 12.5 min. A plateau is therefore present within clinically achievable withdrawal durations, a finding that categorical analysis was unable to resolve and that has not consistently been demonstrated in previous observational work. This pattern was concordant across levels of analysis: at the endoscopist level, where inspection time was measured exclusively in examinations containing no intervention, the gradient likewise attenuated beyond approximately seven minutes, with adenoma detection rising from 14.6% in the lowest quartile of inspection time to 21.0% in the second but only to 23.6% in the highest. The convergence of the point of maximal marginal return with the minimum average withdrawal time of eight minutes recommended in the 2024 ASGE/ACG quality indicators is notable, and our data provide independent real-world support for that recommendation [29]. Equally, the flattening beyond twelve minutes indicates that indefinite prolongation of withdrawal is unlikely to yield proportionate gains, and that the historical six-minute benchmark, while capturing most of the achievable benefit, falls short of the point of maximal return, and the 8 min minimum adopted in 2024 corresponds closely to it.

These findings should not be interpreted as an argument for indefinitely prolonged withdrawal. Withdrawal time is a surrogate for the thoroughness of mucosal inspection rather than a determinant of detection in itself, and our data indicate that it is an incomplete one. Among the 44 endoscopists whose mean inspection time fell between six and eight minutes, adenoma detection ranged from 10.2% to 41.3%, and inspection time accounted for only 19% of the variance in adenoma detection between operators. The substantial residual variation at any given inspection time indicates that how the available time is used matters at least as much as its duration. Techniques of demonstrated value include a second examination of the right colon, either as a second forward view or by retroflexion, which has been shown in randomized trials and meta-analyses to increase right-colon adenoma detection [30,31], water-assisted colonoscopy, and water exchange in particular, which improves mucosal cleanliness during insertion and independently predicts higher adenoma detection [32]; dynamic patient position change during withdrawal to optimize luminal distension segment by segment; and deliberate washing and suctioning of residual debris together with careful examination behind folds and flexures [29]. Withdrawal time is therefore best regarded as a necessary but not sufficient condition for high-quality inspection, and quality-improvement programs should pair a minimum-time standard with training in inspection technique rather than relying on duration alone.

The association between withdrawal duration and adenoma detection remained robust after adjustment for established risk factors. As expected, older age and male sex were independently associated with higher adenoma detection, consistent with the established epidemiology of colorectal neoplasia. No significant interaction was observed between age and withdrawal duration, and stratified estimates were closely similar across age groups, indicating that the relative association with withdrawal duration was similar across the age range and is not confined to patients at higher baseline risk. Similar patterns were observed in both men and women, supporting the generalizability of the findings. The uniformity of the relative association does not, however, imply uniformity of absolute difference.

Although no statistical interaction between age and withdrawal duration was observed, the absolute difference in adenoma detection associated with longer withdrawal increased with age. Extending withdrawal from below six minutes to more than ten minutes was associated with an absolute increase in adenoma detection of 19.1 percentage points in patients under 50 years, 25.2 points in those aged 50 to 59 years, 30.2 points in those aged 60 to 69 years and 31.9 points in those aged 70 years or older, corresponding to approximately 32 additional adenoma-positive examinations per 100 procedures in the oldest group. The constancy of the relative effect alongside a widening absolute difference is the expected consequence of a uniform multiplicative effect acting on a rising baseline prevalence. From a clinical standpoint, absolute yield rather than relative effect determines how many additional lesions are identified, and these data therefore suggest that unhurried inspection is of particular value in older patients, in whom baseline neoplasia prevalence is highest.

Withdrawal time showed only moderate discrimination for adenoma detection, and the operating characteristics presented here indicate that no single threshold is clearly optimal. The Youden index differed only marginally between seven minutes (0.295), 7.68 min (0.302), and eight minutes (0.299), so the precision implied by a two-decimal threshold is not warranted; the Youden-optimal region is better described as approximately seven to eight minutes. Raising the threshold increased the positive predictive value from 26.2% at six minutes to 32.3% at eight minutes and 37.6% at ten minutes, while sensitivity fell from 81.9% to 61.4% and 42.4%, respectively. This pattern accounts for the higher detection rates observed in the longer withdrawal categories and reflects progressive enrichment of the group examined rather than improved discrimination. The threshold observed in the full cohort was also influenced by the inclusion of interventional time: when the analysis was restricted to procedures performed without polypectomy or biopsy, the Youden-optimal threshold fell to 6.43 min with an area under the curve of 0.657. Taken together, these findings indicate that withdrawal time is best regarded as a graded quality measure applied at the level of the endoscopist rather than as a dichotomous criterion applied to individual examinations [29,33,34].

Bowel-preparation score showed a weak negative association with adenoma detection in our models, which is contrary to expectation. This association was present before adjustment and was not monotonic across score categories. Further analysis indicated that it reflects the properties of the recorded score rather than an effect of preparation quality: more than half of the total variance in the score lay between endoscopists rather than between patients, individual operators differed markedly in their scoring conventions, and the association was largely abolished when endoscopist fixed effects were included. At the operator level, mean recorded preparation score was entirely unrelated to adenoma detection. In addition, all scores in this cohort fell within the adequate range, so no comparison with inadequate preparation was possible. These observations indicate that bowel-preparation quality as documented in routine practice may function partly as an operator characteristic rather than as an objective patient-level measurement, and they argue for caution in adjusting for endoscopist-reported preparation scores in observational analyses of colonoscopy quality.

Withdrawal duration was correlated with both lesion-detection outcomes examined. The coefficient for polyp detection exceeded that for adenoma detection, which is the expected pattern given that adenomas constitute a subset of detected polyps and that the two outcomes differ substantially in base rate; the maximum attainable correlation is constrained by the base rate of a binary outcome, so coefficients for outcomes of differing prevalence are not directly comparable. These analyses should therefore be read as confirming the direction and consistency of the association rather than as evidence that prolonged inspection favors the detection of one lesion type over another. We did not analyze the number of polypectomies recorded within the procedure annotation, as this field was populated in only a small minority of examinations and does not provide a valid measure of polyp burden.

The principal limitation of this study is that the recorded withdrawal time includes time spent on biopsy, polypectomy, lesion retrieval, and mucosal cleaning. Our video annotation records the cecal and rectal landmarks but does not record the start and end of individual therapeutic maneuvers; therefore, a procedure-level net inspection time cannot be derived from these data. This is a limitation of the exposure measurement itself and cannot be resolved by covariate adjustment. We addressed it instead by changing the level of analysis: for each endoscopist, inspection time was calculated exclusively from examinations in which no polyp was detected and no tissue was sampled, so that the exposure and the outcome are derived from disjoint sets of procedures and the within-procedure reverse-causation mechanism cannot operate. This is also the quantity specified by the ASGE/ACG quality indicator, which is defined as the average withdrawal time of an endoscopist in normal colonoscopies performed without biopsy or polypectomy.

This study has several strengths, including its large sample size, its multicenter design, the use of automatically recorded procedural timings rather than manually entered values, and the availability of endoscopist identifiers permitting analysis at the operator level. Several limitations should nonetheless be acknowledged. First, the retrospective observational design prevents causal inference and leaves the possibility of residual confounding. Second, and most importantly, the recorded withdrawal time included time spent performing biopsy, polypectomy, lesion retrieval, and mucosal cleaning; the video annotation records the cecal and rectal landmarks but not the duration of individual interventions, so a procedure-level net inspection time could not be derived. We addressed this by measuring inspection time at the level of the endoscopist in examinations containing no intervention, and by three procedure-level sensitivity analyses, all of which were concordant with the principal findings, but the exposure measured at the level of the individual procedure remains susceptible to reverse causation. Third, endoscopist age, seniority and years of experience were not available within the anonymized dataset, and residual confounding by operator characteristics therefore cannot be excluded, since more experienced endoscopists may both inspect more thoroughly and detect more lesions; annual procedure volume, the only operator characteristic derivable from our data, did not attenuate the association between inspection time and adenoma detection, but this does not exclude confounding by experience itself. Fourth, adenoma detection was ascertained from coded histopathology records and sessile serrated lesions could not be identified separately; given their recognized association with longer inspection times, our findings may underestimate the relationship with clinically important non-adenomatous neoplasia. The adenoma detection rate observed in this cohort was lower than the minimum recommended by current quality indicators, including within the screening subgroup. This most probably reflects ascertainment from coded histopathology in which sessile serrated lesions were not separately identified, together with the inclusion of examinations performed below the usual screening age and across mixed indications. Our findings should therefore be read as describing the relationship between inspection duration and detection within this cohort rather than as a benchmark of endoscopic performance. Fifth, bowel-preparation scores ranged only from 6 to 9 on the BBPS and were bimodally distributed, so that all procedures were performed under adequate preparation and the influence of inadequate preparation could not be evaluated; our findings should not be extrapolated to examinations performed under suboptimal preparation. Preparation quality was moreover documented by the performing endoscopist rather than by an independent assessor, and more than half of the variance in the recorded score lay between operators rather than between patients; the score should therefore be regarded as only a partial measure of true preparation quality, and adjustment for it may be incomplete. Sixth, a cecal landmark was confirmed by the automated annotation in 93.2% of procedures; examination of the remainder indicated that the withdrawal interval had defaulted to approximately the whole procedure duration when the landmark was not identified, while total procedure duration was indistinguishable from that of confirmed examinations, so that these represent annotation failure with an overestimated withdrawal time rather than incomplete examinations. All principal and supportive analyses were repeated with the cohort restricted to confirmed examinations, with materially unchanged results. Finally, information on the use of advanced imaging techniques and on lesion morphology was not available.

In conclusion, inspection time measured at the level of the endoscopist in examinations containing no therapeutic intervention was associated with adenoma detection in this large multicenter colonoscopy cohort, a design that eliminates the within-procedure mechanism by which detection and resection lengthen the recorded withdrawal time of the same examination; the same gradient was evident at the procedure level, where recorded withdrawal duration additionally incorporates time spent on therapeutic intervention. The association extended beyond the historical six-minute benchmark and was consistent across age and sex strata, although flexible modeling of recorded withdrawal duration demonstrated that the marginal return peaked at approximately eight minutes and diminished substantially thereafter, and the endoscopist-level analysis showed corresponding attenuation of the gradient beyond approximately seven minutes. The absolute difference in adenoma detection associated with longer withdrawal was greatest in older patients. These findings support the concept that withdrawal time is a graded marker of inspection quality rather than a binary threshold, and they provide real-world support for the recent revision of the recommended minimum average withdrawal time from six to eight minutes [29]. Withdrawal time should nonetheless be understood as a surrogate for careful mucosal inspection rather than an end in itself, and quality-improvement efforts should address inspection technique alongside procedural duration. Prospective studies incorporating operator experience and separating net inspection time from interventional time are required to determine whether targeted optimization of withdrawal practice can further improve adenoma detection and colorectal cancer prevention.

## Figures and Tables

**Table 1:** Baseline characteristics of the study cohort.

Variable	Value	*n* (%)
Total colonoscopies	31,780	—
Age, years—median (IQR)	60.4 (53.5–68.3)	—
Male sex	—	14,909 (46.9)
Female sex	—	16,871 (53.1)
Withdrawal time, min—median (IQR)	6.83 (4.80–9.53)	—
Boston Bowel-Preparation Scale—median (IQR)	7 (6–9)	—
BBPS 6	—	15,709 (49.4)
BBPS 7	—	4430 (13.9)
BBPS 8	—	3589 (11.3)
BBPS 9	—	8052 (25.4)
**Colonoscopy indication**		
Screening	—	8229 (25.9)
Symptomatic or diagnostic	—	7647 (24.1)
Polyp surveillance	—	4142 (13.0)
Family history of colorectal cancer	—	3672 (11.6)
Positive fecal occult blood	—	1903 (6.0)
Other or unspecified	—	3815 (12.0)
Not recorded	—	2372 (7.4)
**Study site**		
Ra’anana	—	7646 (24.1)
Haifa	—	6399 (20.1)
Ramat HaChayal	—	5794 (18.2)
Holon	—	4304 (13.5)
Ashdod	—	3366 (10.6)
Jerusalem	—	2121 (6.7)
Beer Sheva	—	1887 (5.9)
Not recorded	—	263 (0.9)
Polyp detected	—	13,207 (41.6)
Adenoma detected	—	6252 (19.7)

Continuous and ordinal variables are presented as median with interquartile range (IQR) and categorical variables as number (percentage). Age, withdrawal time, and Boston Bowel-Preparation Scale (BBPS) score were non-normally distributed. The BBPS distribution was bimodal, with 74.7% of procedures scored either 6 or 9; no procedure had a score below 6. Colonoscopy indication was classified hierarchically from the structured presenting-complaint entries recorded in the clinical endoscopy report, with precedence given to positive fecal occult blood testing, then polyp surveillance, then family history, then screening, then symptomatic presentation; an indication could be assigned in 25,593 procedures (80.5%). All procedures were performed between 1 January and 8 December 2025 across seven gastroenterology units of Assuta Medical Centers.

**Table 2:** Adenoma and polyp detection across ordered withdrawal-time categories.

Withdrawal-Time Category	*N*	Mean Withdrawal Time, min	Adenoma Detection, *n* (%)	Polyp Detection, *n* (%)
<6 min	12,338	4.04	1147 (9.30)	2952 (23.93)
6–8 min	7600	6.93	1276 (16.79)	2872 (37.79)
8–10 min	4821	8.92	1186 (24.60)	2543 (52.75)
>10 min	7021	14.37	2643 (37.64)	4840 (68.94)

Detection rates are presented as the number and percentage of examinations in which at least one lesion was identified. Category boundaries are right-inclusive, so that a value falling exactly on a boundary is assigned to the lower category; an examination of exactly 6.00 min is therefore counted in the <6 min category. The Cochran–Armitage test was used to assess ordered trends across progressively longer withdrawal-time categories: adenoma detection rate, χ^2^ for trend = 2354.12 (*p* < 0.001); polyp detection rate, χ^2^ for trend = 4035.04 (*p* < 0.001). Adenoma detection was defined as at least one histologically confirmed adenoma and polyp detection as at least one colorectal polyp identified at endoscopy.

**Table 3:** Association between withdrawal time and adenoma detection in multivariable logistic regression analysis.

Withdrawal-Time Specification	Adjusted OR (95% CI)	Cluster-Robust OR (95% CI)	*p* Value
Continuous, per additional minute	1.14 (1.13–1.15)	1.14 (1.13–1.16)	<0.001
Categorical, <6 min	Reference	Reference	—
6–8 min	1.93 (1.77–2.10)	1.93 (1.69–2.19)	<0.001
8–10 min	3.07 (2.80–3.36)	3.07 (2.66–3.54)	<0.001
>10 min	5.54 (5.12–5.99)	5.54 (4.79–6.40)	<0.001

Multivariable logistic regression evaluating the association between withdrawal time and adenoma detection, adjusted for age, sex and Boston Bowel-Preparation Scale score (entered as a categorical covariate with a score of 6 as the reference). Continuous and categorical specifications were fitted as separate models. Cluster-robust confidence intervals were obtained using the Huber–White sandwich estimator with the endoscopist as the clustering unit; point estimates are identical to those of the model-based analysis. The full covariate output is presented in Supplementary Table S1. CI, confidence interval; OR, odds ratio.

**Table 4:** Correlation between withdrawal duration and lesion-detection outcomes.

Outcome	Point-Biserial r	Spearman ρ	*p* Value
Polyp detection (binary)	0.346	0.364	<0.001
Adenoma detection (binary)	0.279	0.276	<0.001

Coefficients for binary outcomes are point-biserial correlations; Spearman coefficients are presented in parallel. Both associations were statistically significant. The difference in magnitude between the two outcomes should not be interpreted as evidence of selective enrichment, since adenoma detection is a subset of polyp detection and the two outcomes have substantially different base rates (41.6% and 19.7%, respectively), which constrains the maximum attainable correlation. The number of polypectomies recorded within the procedure annotation was not analyzed, as this field was populated in only 3.3% of examinations and does not provide a valid measure of polyp burden.

**Table 5:** Operating characteristics of withdrawal time for the prediction of adenoma detection.

Cut-Off, min	Procedures Above Cut-Off, *n* (%)	Sensitivity, %	Specificity, %	Youden Index	PPV, %	NPV, %	LR+
6.00	19,529 (61.5)	81.9	43.6	0.254	26.2	90.8	1.45
7.00	15,253 (48.0)	71.7	57.8	0.295	29.4	89.3	1.70
7.68	12,855 (40.5)	64.7	65.5	0.302	31.5	88.3	1.88
8.00	11,888 (37.4)	61.4	68.5	0.299	32.3	87.9	1.95
9.00	9130 (28.7)	51.3	76.8	0.281	35.1	86.6	2.21
10.00	7045 (22.2)	42.4	82.8	0.251	37.6	85.4	2.46
12.00	4316 (13.6)	28.3	90.0	0.184	41.1	83.7	2.84

The area under the receiver operating characteristic curve is a global measure of discrimination derived from the entire curve and does not vary with the chosen cut-off; for withdrawal time as a predictor of adenoma detection, it was 0.701 (95% confidence interval 0.693 to 0.708). Cut-offs are shown at clinically relevant values together with the Youden-optimal value of 7.68 min. The Youden index was maximal at 7.68 min but differed only marginally at 7.00 and 8.00 min, indicating that no single threshold is clearly superior. When the analysis was restricted to the 15,487 procedures performed without polypectomy or biopsy, the area under the curve was 0.657, and the Youden-optimal threshold was 6.43 min. LR+, positive likelihood ratio; NPV, negative predictive value; and PPV, positive predictive value.

**Table 6:** Adenoma and polyp detection according to withdrawal-time category, stratified by sex.

Sex	Withdrawal-Time Category	*N*	Mean Withdrawal Time, min	Adenoma Detection, *n* (%)	Polyp Detection, *n* (%)
Female	<6 min	7112	4.01	526 (7.40)	1467 (20.63)
	6–8 min	4110	6.92	561 (13.65)	1354 (32.94)
	8–10 min	2387	8.91	512 (21.45)	1128 (47.26)
	>10 min	3262	14.25	1055 (32.34)	2046 (62.72)
Male	<6 min	5226	4.08	621 (11.88)	1485 (28.42)
	6–8 min	3490	6.94	715 (20.49)	1518 (43.50)
	8–10 min	2434	8.93	674 (27.69)	1415 (58.13)
	>10 min	3759	14.48	1588 (42.25)	2794 (74.33)

Detection rates are presented as the number and percentage of examinations within each stratum in which at least one lesion was identified. Category boundaries are right-inclusive, so that a value falling exactly on a boundary is assigned to the lower category; an examination of exactly 6.00 min is therefore counted in the <6 min category. Cochran–Armitage tests for trend across ordered withdrawal-time categories were significant in both sexes: in women, χ^2^ = 1109.15 for adenoma detection and 1893.26 for polyp detection; in men, χ^2^ = 1123.83 and 1973.03, respectively (all *p* < 0.001). The cohort comprised 16,871 women and 14,909 men.

**Table 7:** Adenoma and polyp detection according to withdrawal-time category, stratified by age group.

Age Group, Years	Withdrawal-Time Category	*N*	Mean Withdrawal Time, min	Adenoma Detection, *n* (%)	Polyp Detection, *n* (%)
<50	<6 min	1332	3.97	55 (4.13)	213 (15.99)
	6–8 min	762	6.88	70 (9.19)	207 (27.17)
	8–10 min	454	8.89	69 (15.20)	184 (40.53)
	>10 min	565	13.92	131 (23.19)	296 (52.39)
50–59	<6 min	4954	4.07	366 (7.39)	983 (19.84)
	6–8 min	2921	6.93	413 (14.14)	995 (34.06)
	8–10 min	1896	8.93	396 (20.89)	898 (47.36)
	>10 min	2527	14.33	824 (32.61)	1634 (64.66)
60–69	<6 min	3704	4.09	395 (10.66)	1038 (28.02)
	6–8 min	2446	6.93	459 (18.77)	995 (40.68)
	8–10 min	1486	8.91	414 (27.86)	847 (57.00)
	>10 min	2339	14.36	956 (40.87)	1688 (72.17)
≥70	<6 min	2348	3.94	331 (14.10)	718 (30.58)
	6–8 min	1471	6.94	334 (22.71)	675 (45.89)
	8–10 min	985	8.93	307 (31.17)	614 (62.34)
	>10 min	1590	14.61	732 (46.04)	1222 (76.86)

Detection rates are presented as the number and percentage of examinations within each stratum in which at least one lesion was identified. Category boundaries are right-inclusive, so that a value falling exactly on a boundary is assigned to the lower category; an examination of exactly 6.00 min is therefore counted in the <6 min category. Age groups comprised 3113 patients aged under 50 years, 12,298 aged 50–59 years, 9975 aged 60–69 years and 6394 aged 70 years or older. Cochran–Armitage tests for trend across ordered withdrawal-time categories were significant in every age stratum: for adenoma detection, χ^2^ = 165.64, 809.13, 791.48 and 509.24 respectively; for polyp detection, χ^2^ = 290.37, 1555.11, 1224.57 and 882.00 (all *p* < 0.001). The absolute difference in adenoma detection between the shortest and longest withdrawal categories increased with age, from 19.1 percentage points in patients under 50 years to 25.2, 30.2 and 31.9 percentage points in the successive age strata.

**Table 8:** Adenoma and polyp detection across finely stratified withdrawal-time intervals.

Withdrawal-Time Interval	*N*	Mean Withdrawal Time, min	Adenoma Detection, *n* (%)	Polyp Detection, *n* (%)
0–4 min	5490	2.78	379 (6.90)	1069 (19.47)
4–6 min	6848	5.06	768 (11.21)	1883 (27.50)
6–8 min	7600	6.93	1276 (16.79)	2872 (37.79)
8–10 min	4821	8.92	1186 (24.60)	2543 (52.75)
10–15 min	4877	11.97	1670 (34.24)	3159 (64.77)
>15 min	2144	19.82	973 (45.38)	1681 (78.40)

Detection rates are presented as the number and percentage of examinations in which at least one lesion was identified. Interval boundaries are right-inclusive, so that a value falling exactly on a boundary is assigned to the lower interval; the six intervals together comprise the full analytic cohort of 31,780 procedures. Cochran–Armitage tests for trend across ordered intervals were highly significant for both outcomes (adenoma detection, χ^2^ = 2458.95; polyp detection, χ^2^ = 4185.38; *p* < 0.001). The longest recorded withdrawal time was 63.3 min; no upper restriction was applied.

**Table 9:** Endoscopist-level inspection time and lesion detection.

Inspection Time Quartile	Endoscopists, *n*	Procedures, *n*	Mean Inspection Time, min	Inspection Time Range, min	ADR, %	PDR, %
Q1	27	7363	3.74	2.02–4.81	14.6	37.3
Q2	27	7199	5.84	4.90–6.57	21.0	41.9
Q3	26	7242	7.05	6.61–7.61	22.2	42.8
Q4	27	4989	9.14	7.69–13.34	23.6	45.5

Inspection time was defined for each endoscopist as the mean withdrawal time across colonoscopies in which no polyp was detected and no tissue was sampled (n = 14,611 examinations). ADR and PDR were calculated across all procedures performed by each endoscopist. Analyses were restricted to endoscopists contributing ≥100 procedures and ≥30 negative examinations (107 endoscopists; 26,793 procedures). Spearman correlation between inspection time and ADR, ρ = 0.46 (*p* < 0.001); between inspection time and PDR, ρ = 0.33 (*p* < 0.001). Endoscopists with a mean inspection time ≥6 min had a higher ADR than those below this benchmark (21.8% vs. 15.7%; *p* < 0.001). ADR, adenoma detection rate; PDR, polyp detection rate.

**Table 10:** Sensitivity analysis of endoscopist-level inspection time after exclusion of endoscopists with a mean inspection time below 3 min.

Inspection Time Quartile	Endoscopists, *n*	Procedures, *n*	Mean Inspection Time, min	ADR, %	PDR, %
Q1	26	7857	3.98	16.8	39.6
Q2	26	6363	5.94	19.5	40.3
Q3	26	7336	7.09	23.0	43.8
Q4	26	4766	9.19	22.8	44.5

Three of 107 endoscopists (471 procedures; 1.8% of the analytic sample) had a mean inspection time below 3 min, a duration incompatible with complete colonic examination and most probably reflecting sigmoidoscopy, incomplete procedures, or annotation error. After their exclusion (104 endoscopists; 26,322 procedures), the association was preserved (Spearman ρ = 0.42; *p* < 0.001), and endoscopists reaching the historical 6 min benchmark maintained a higher ADR than those below it (21.8% vs. 16.2%; *p* < 0.001). ADR, adenoma detection rate; PDR, polyp detection rate.

**Table 11:** Polyp detection across withdrawal-time categories in procedures performed without polypectomy or biopsy.

Withdrawal-Time Category	*N*	Mean Withdrawal Time, min	PDR, %	Adjusted OR (95% CI)	*p* Value
<6 min	7974	3.94	3.44	Reference	—
6–8 min	3891	6.88	5.11	1.50 (1.24–1.81)	<0.001
8–10 min	1849	8.86	8.87	2.73 (2.23–3.34)	<0.001
>10 min	1773	13.37	13.48	4.29 (3.57–5.15)	<0.001

Analysis restricted to the 15,487 procedures in which neither polypectomy nor biopsy was performed and in which the recorded withdrawal time therefore contained no interventional component. Adenoma detection rate cannot be calculated in this subgroup because histological confirmation requires tissue sampling. Odds ratios were adjusted for age, sex and Boston Bowel-Preparation Scale score. Cochran–Armitage test for trend, χ^2^ = 300.2 (*p* < 0.001). Modeled as a continuous variable, each additional minute of withdrawal time was associated with an adjusted OR of 1.14 (95% CI 1.12–1.15; *p* < 0.001); the estimate was unchanged when standard errors were clustered on the endoscopist (1.14, 95% CI 1.10–1.17). CI, confidence interval; OR, odds ratio; and PDR, polyp detection rate.

**Table 12:** Fine-interval analysis of polyp detection in procedures performed without polypectomy or biopsy.

Withdrawal Time Interval	*N*	Mean Withdrawal Time, min	PDR, %
0–4 min	3830	2.76	3.03
4–6 min	4144	5.03	3.81
6–8 min	3891	6.88	5.11
8–10 min	1849	8.86	8.87
10–15 min	1395	11.82	11.83
>15 min	378	19.09	19.58

Analysis restricted to the 15,487 procedures performed without polypectomy or biopsy. Cochran–Armitage test for trend, χ^2^ = 298.4 (*p* < 0.001). The graded increase in polyp detection persisted in the complete absence of interventional time. In this subgroup, receiver operating characteristic analysis yielded an area under the curve of 0.657 and an optimal threshold of 6.43 min, compared with 7.68 min in the full cohort. PDR, polyp detection rate.

**Table 13:** Withdrawal time and lesion detection according to colonoscopy indication.

Indication	*n*	Median Withdrawal Time, min	ADR, %	PDR, %
Positive fecal occult blood	1903	8.10	38.26	58.01
Polyp surveillance	4142	7.85	31.14	57.92
Family history of colorectal cancer	3672	6.83	17.32	36.08
Screening	8229	6.48	15.45	35.82
Symptomatic or diagnostic	7647	6.73	15.29	36.28
Other or unspecified	3815	7.53	19.53	42.99
Not recorded	2372	4.78	17.41	42.88

Indications were classified hierarchically from the structured presenting-complaint entries recorded in the clinical endoscopy report. ADR, adenoma detection rate; PDR, polyp detection rate.

**Table 14:** Association between withdrawal time and adenoma detection under progressive adjustment and within restricted populations.

Model or Population	*n*	Adjusted OR Per Minute (95% CI)
Age, sex, bowel preparation	31,780	1.141 (1.134–1.148)
+Colonoscopy indication	31,780	1.135 (1.128–1.143)
+Institution	31,780	1.142 (1.135–1.150)
+Institution and endoscopist	31,780	1.183 (1.173–1.192)
Restricted to screening	8229	1.159 (1.143–1.174)
Restricted to screening and family history	11,901	1.148 (1.136–1.161)
Restricted to confirmed cecal landmark	29,607	1.175 (1.166–1.184)

Institution and endoscopist were entered as fixed effects. All models were adjusted for age, sex and bowel-preparation quality; restricted analyses were additionally adjusted within stratum. CI, confidence interval; OR, odds ratio. Estimates are presented to three decimal places so that differences between successive adjustment sets remain visible; all associations were significant at *p* < 0.001.

**Table 15:** Detection of advanced histology across withdrawal-time categories.

Withdrawal Time	*N*	Advanced Histology, *n* (%)	Rate, %
<6 min	12,338	69	0.56
6–8 min	7600	71	0.93
8–10 min	4821	67	1.39
>10 min	7021	307	4.37

Advanced histology was defined as tubulovillous or villous architecture, dysplasia within an adenoma, or invasive carcinoma within the coded histopathology record. Adjusted odds ratio 1.127 (95% CI 1.114–1.140) per additional minute, adjusted for age, sex and bowel-preparation quality; *p* < 0.001.

## Data Availability

The original contributions presented in this study are included in the article. Further inquiries can be directed to the corresponding author.

## Supplementary Materials

The following supporting information can be downloaded at: https://www.mdpi.com/article/10.3390/diagnostics16172693/s1, Table S1. Covariate estimates from the multivariable logistic regression models for adenoma detection. Table S2. Estimates for withdrawal time under alternative specifications of clustering and confounding. Table S3. Stratified estimates of the association between withdrawal time and adenoma detection. Table S4. Sex- and age-stratified polyp detection across withdrawal-time categories in procedures performed without polypectomy or biopsy. Table S5. Summary of sensitivity analyses addressing the contribution of interventional time to the observed association. Table S6. Association between endoscopist mean inspection time, assigned to each of that endoscopist’s procedures, and patient-level adenoma detection. Table S7. Principal and supportive analyses repeated with the cohort restricted to procedures with a confirmed cecal landmark. Figure S1. Study flow diagram. Colonoscopies recorded in the clinical endoscopy database were linked to the cor-responding video-annotation records by procedure identifier and validated on sex, age, and procedure date. The final analytic cohort comprised 31,780 colonoscopies performed in 31,690 patients across seven endoscopy units. The lower panel shows derivation of the endoscopist-level analytic sample, in which inspection time was calculated from examinations containing no polyp and no tissue sampling.
