# Supplementary material for: Association Between Colonoscopy Withdrawal Time and Adenoma Detection: Evidence of a Continuous Dose–Response Relationship
Source: Diagnostics (Basel). 2026 Aug 24;16(17):2693. doi: 10.3390/diagnostics16172693 (PMC13564573; doi:10.3390/diagnostics16172693)
Supplement: Supplementary file 1 [file diagnostics-16-02693-s001.zip › diagnostics-4419271-supplementary.pdf]

diagnostics

MDPI

Supplementary Tables

Table S1. Covariate estimates from the multivariable logistic regression models for adenoma detection.

Covariate	Adjusted OR (95% CI)	Cluster-robust OR (95% CI)	p value
Age, per additional year	1.04 (1.03-1.04)	1.04 (1.03-1.04)	<0.001
Male sex (reference: female)	1.57 (1.48-1.66)	1.57 (1.47-1.67)	<0.001
BBPS 7 (reference: BBPS 6)	0.90 (0.83-0.99)	0.90 (0.76-1.07)	0.027
BBPS 8 (reference: BBPS 6)	0.94 (0.86-1.04)	0.94 (0.80-1.12)	0.240
BBPS 9 (reference: BBPS 6)	0.90 (0.83-0.96)	0.90 (0.74-1.09)	0.003

Covariate estimates from the categorical withdrawal-time model presented in Table 3. p values correspond to the model-based analysis. None of the Boston Bowel Preparation Scale (BBPS) categories remained statistically significant when standard errors were clustered on the endoscopist, and the association across categories was non-monotonic; these estimates should therefore not be interpreted as evidence of a graded effect of preparation quality on adenoma detection. CI, confidence interval; OR, odds ratio.

Table S2. Estimates for withdrawal time under alternative specifications of clustering and confounding.

Specification	Adjusted OR per minute (95% CI)	Standard errors
Principal model	1.141 (1.134-1.148)	Model-based
Principal model, cluster-robust standard errors	1.141 (1.125-1.157)	Cluster-robust, endoscopist
+ institution fixed effects	1.142 (1.135-1.150)	Model-based
+ institution and endoscopist fixed effects, with indication	1.183 (1.173-1.192)	Model-based
+ institution and endoscopist fixed effects, without indication	1.191 (1.181-1.200)	Model-based

All models were adjusted for age, sex and bowel preparation quality; models from the second row onwards were additionally adjusted for colonoscopy indication except where stated. Cluster-robust standard errors were obtained using the Huber–White sandwich estimator with the endoscopist as the clustering unit; point estimates are identical to those of the corresponding model-based analysis.

Table S3. Stratified estimates of the association between withdrawal time and adenoma detection.

Stratum	N	Adjusted OR per additional minute (95% CI)	Cluster-robust 95% CI
Age <50 years	3,113	1.148 (1.121-1.176)	1.118-1.180
Age 50-59 years	12,298	1.139 (1.127-1.151)	1.120-1.159
Age 60-69 years	9,975	1.144 (1.132-1.156)	1.125-1.164
Age ≥70 years	6,394	1.136 (1.123-1.150)	1.116-1.157
Women	16,871	1.147 (1.137-1.157)	—
Men	14,909	1.135 (1.126-1.145)	—

Separate multivariable logistic regression models were fitted within each stratum. Age-stratified models were adjusted for sex and Boston Bowel Preparation Scale score; sex-stratified models were adjusted for age and Boston Bowel Preparation Scale score. Estimates were closely similar across all strata, and the confidence intervals overlapped substantially; the ratio of the estimates in the youngest and oldest age strata was 0.990. Cluster-robust intervals use the endoscopist as the clustering unit. CI, confidence interval; OR, odds ratio.

Table S4. Sex- and age-stratified polyp detection across withdrawal-time categories in procedures performed without polypectomy or biopsy.

Stratum	N	<6 min, %	6-8 min, %	8-10 min, %	>10 min, %	p for trend
Female	8,800	2.86	4.36	8.54	10.99	<0.001
Male	6,687	4.26	6.08	9.25	16.27	<0.001
Age <50 y	1,751	2.74	3.46	6.37	6.97	<0.001
Age 50–59 y	6,536	2.60	4.28	6.48	10.72	<0.001
Age 60–69 y	4,532	4.57	5.35	9.90	15.33	<0.001
Age ≥70 y	2,668	4.07	7.88	15.23	20.69	<0.001

Values are polyp detection rates within each withdrawal-time category. Analysis restricted to the 15,487 procedures performed without polypectomy or biopsy. Trends were assessed with the Cochran–Armitage test. The graded association between withdrawal duration and polyp detection was preserved in every demographic stratum.

Table S5. Summary of sensitivity analyses addressing the contribution of interventional time to the observed association.

Analysis	Analytic sample	Outcome	Effect estimate	p value
Main procedure-level model	31,780 procedures	ADR	aOR 1.14 (1.13-1.15) per min	<0.001
Clustered standard errors (endoscopist)	31,780 procedures	ADR	aOR 1.14 (1.13-1.16) per min	<0.001
Coded polypectomies excluded	22,997 procedures	ADR	aOR 1.19 (1.18-1.20) per min	<0.001
Procedures without polypectomy or biopsy	15,487 procedures	PDR	aOR 1.14 (1.12-1.15) per min	<0.001
Endoscopist-level inspection time	107 endoscopists	ADR	Spearman ϱ = 0.46	<0.001
Endoscopist-level, <3 min excluded	104 endoscopists	ADR	Spearman ϱ = 0.42	<0.001

All models were adjusted for age, sex, and Boston Bowel Preparation Scale score. Concordance of the six analyses indicates that the association between withdrawal duration and lesion detection is not attributable to the inclusion of interventional time within the recorded withdrawal duration. aOR, adjusted odds ratio; ADR, adenoma detection rate; PDR, polyp detection rate.

Table S6. Association between endoscopist mean inspection time, assigned to each of that endoscopist's procedures, and patient-level adenoma detection.

Model or population	Procedures, n	Adjusted OR per additional minute (95% CI)
Age, sex, bowel preparation	26,793	1.086 (1.046-1.129)
+ colonoscopy indication	26,793	1.090 (1.045-1.136)
+ colonoscopy indication and institution	26,793	1.086 (1.046-1.129)
Restricted to screening	6,824	1.121 (1.068-1.177)
Restricted to screening and family history	9,947	1.098 (1.055-1.142)

Each endoscopist's mean withdrawal time, calculated exclusively from colonoscopies in which no polyp was detected and no tissue was sampled, was assigned as an exposure to every procedure performed by that endoscopist. Analyses were restricted to the 107 endoscopists contributing at least 100 procedures and at least 30 negative examinations, defined as examinations in which no polyp was detected and no tissue was sampled. All confidence intervals are cluster-robust with the endoscopist as the clustering unit; because the exposure is constant

within endoscopist, these intervals are appreciably wider than the corresponding model-based intervals and reflect the 107 independent units of exposure. All associations were significant at $p < 0.001$. CI, confidence interval; OR, odds ratio.

Table S7. Principal and supportive analyses repeated with the cohort restricted to procedures with a confirmed cecal landmark.

Analysis	Full cohort	Confirmed cecal landmark
Endoscopists / procedures	107 / 26,793	101 / 25,003
Negative examinations	14,611	13,500
Endoscopist-level correlation (Spearman ϱ)	0.463	0.456
Assigned exposure, fully adjusted	1.086 (1.046-1.129)	1.091 (1.050-1.134)
Assigned exposure, screening only	1.121 (1.068-1.177)	1.118 (1.060-1.180)
Procedure-level recorded withdrawal time	1.141 (1.125-1.157)	1.175 (1.152-1.198)

Values are adjusted odds ratios per additional minute with 95% confidence intervals unless stated otherwise. Assigned-exposure models were adjusted for age, sex, bowel preparation quality, colonoscopy indication and institution, with cluster-robust confidence intervals using the endoscopist as the clustering unit; the screening-only model was adjusted for age, sex, bowel preparation quality and institution. Procedure-level models were adjusted for age, sex and bowel preparation quality. In procedures without a confirmed cecal landmark the recorded withdrawal time defaulted to approximately the whole procedure duration, and the higher procedure-level estimate under restriction reflects removal of these inflated values. All associations were significant at $p < 0.001$. CI, confidence interval; OR, odds ratio.

Supplementary Figure

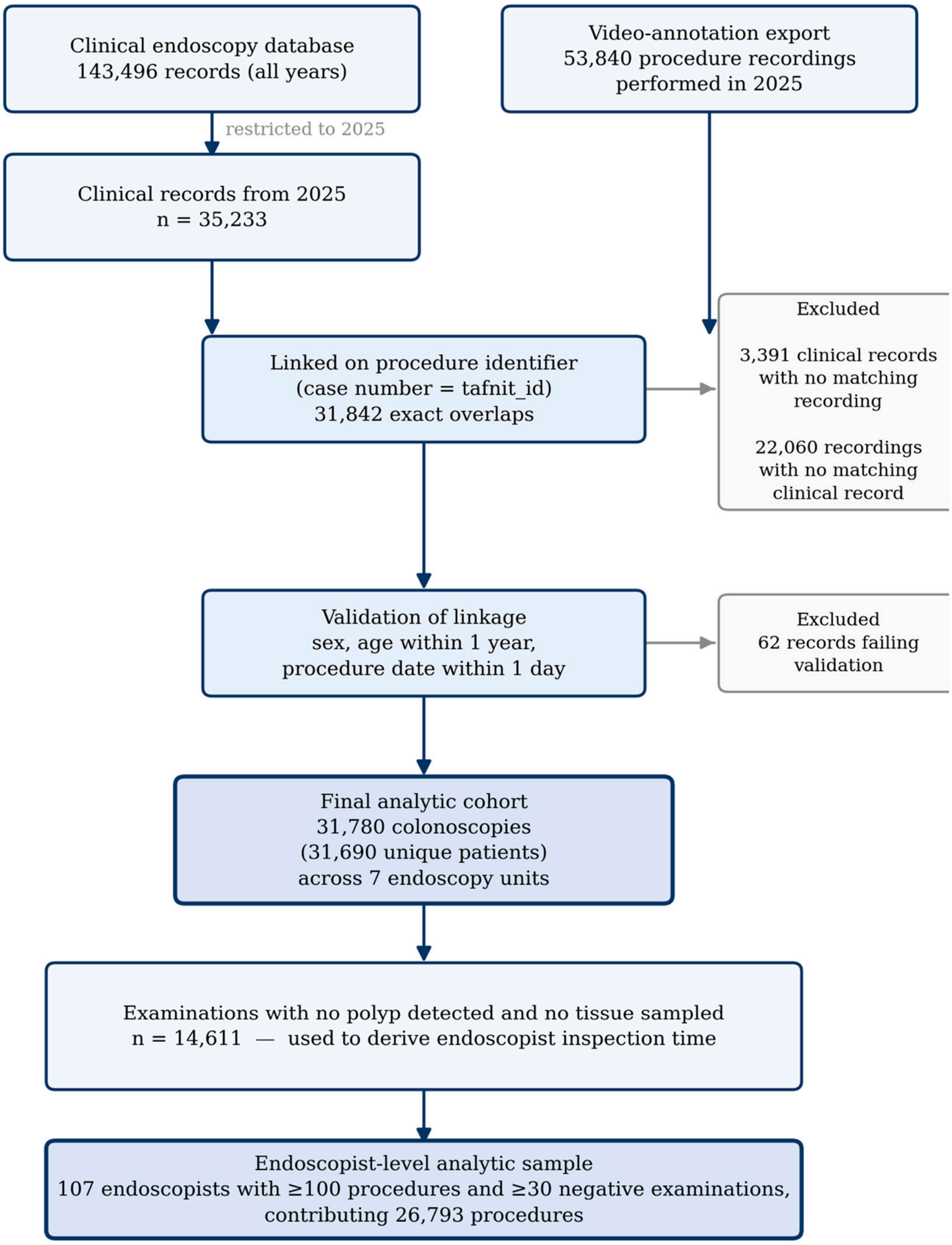

Supplementary Figure S1. Study flow diagram. Colonoscopies recorded in the clinical endoscopy database were linked to the corresponding video-annotation records by procedure identifier and validated on sex, age, and procedure date. The final analytic cohort comprised 31,780 colonoscopies performed in 31,690 patients across seven endoscopy units. The lower panel shows derivation of the endoscopist-level analytic sample, in which inspection time was calculated from examinations containing no polyp and no tissue sampling.
